# HuR promotes castration-resistant prostate cancer progression by altering ERK5 activation via posttranscriptional regulation of BCAT1

**DOI:** 10.1186/s12967-024-04970-w

**Published:** 2024-02-18

**Authors:** Hang You, Guojing Song, Zhizhen Xu, Saipeng Chen, Wenhao Shen, Heting Liu, Bingqian Deng, Jun Li, Gang Huang

**Affiliations:** 1 Department of Urologic Oncology Surgery, Chongqing University Cancer Hospital, HanYu Road 181, Chongqing, 400030 China; 2 School of Medicine, Chongqing University, Chongqing, 400030 China; 3 Department of Biochemistry and Molecular Biology, College of Basic Medical Science, Army Medical University, GaoTanYan Main Street 30, Chongqing, 400038 China; 4 Department of Urology, Southwest Hospital, Amy Medical University, Chongqing, 400038 China

**Keywords:** Human antigen R, Branched-chain amino transferase 1, Castration-resistant prostate cancer, ERK5, KH-3

## Abstract

**Background:**

Castration-resistant prostate cancer (CRPC) is refractory to hormone treatment, and the underlying mechanism has not been fully elucidated. This study aimed to clarify the role and mechanism of Human antigen R (HuR) as a therapeutic target for CRPC progression.

**Methods:**

HuR was knocked out by Cas9 or inhibited by the HuR-specific inhibitor KH-3 in CRPC cell lines and in a mouse xenograft model. The effects of HuR inhibition on tumour cell behaviors and signal transduction were examined by proliferation, transwell, and tumour xenograft assays. Posttranscriptional regulation of BCAT1 by HuR was determined by half-life and RIP assays.

**Results:**

HuR knockout attenuated the proliferation, migration, and invasion of PC3 and DU145 cells in vitro and inhibited tumour progression in vivo. Moreover, BCAT1 was a direct target gene of HuR and mediated the oncogenic effect of HuR on CRPC. Mechanistically, HuR directly interacted with *BCAT1* mRNA and upregulated BCAT1 expression by increasing the stability and translation of BCAT1, which activated ERK5 signalling. Additionally, the HuR-specific inhibitor KH-3 attenuated CRPC progression by disrupting the HuR-BCAT1 interaction.

**Conclusions:**

We confirmed that the HuR/BCAT1 axis plays a crucial role in CRPC progression and suggest that inhibiting the HuR/BCAT1 axis is a promising therapeutic approach for suppressing CRPC progression.

**Graphical Abstract:**

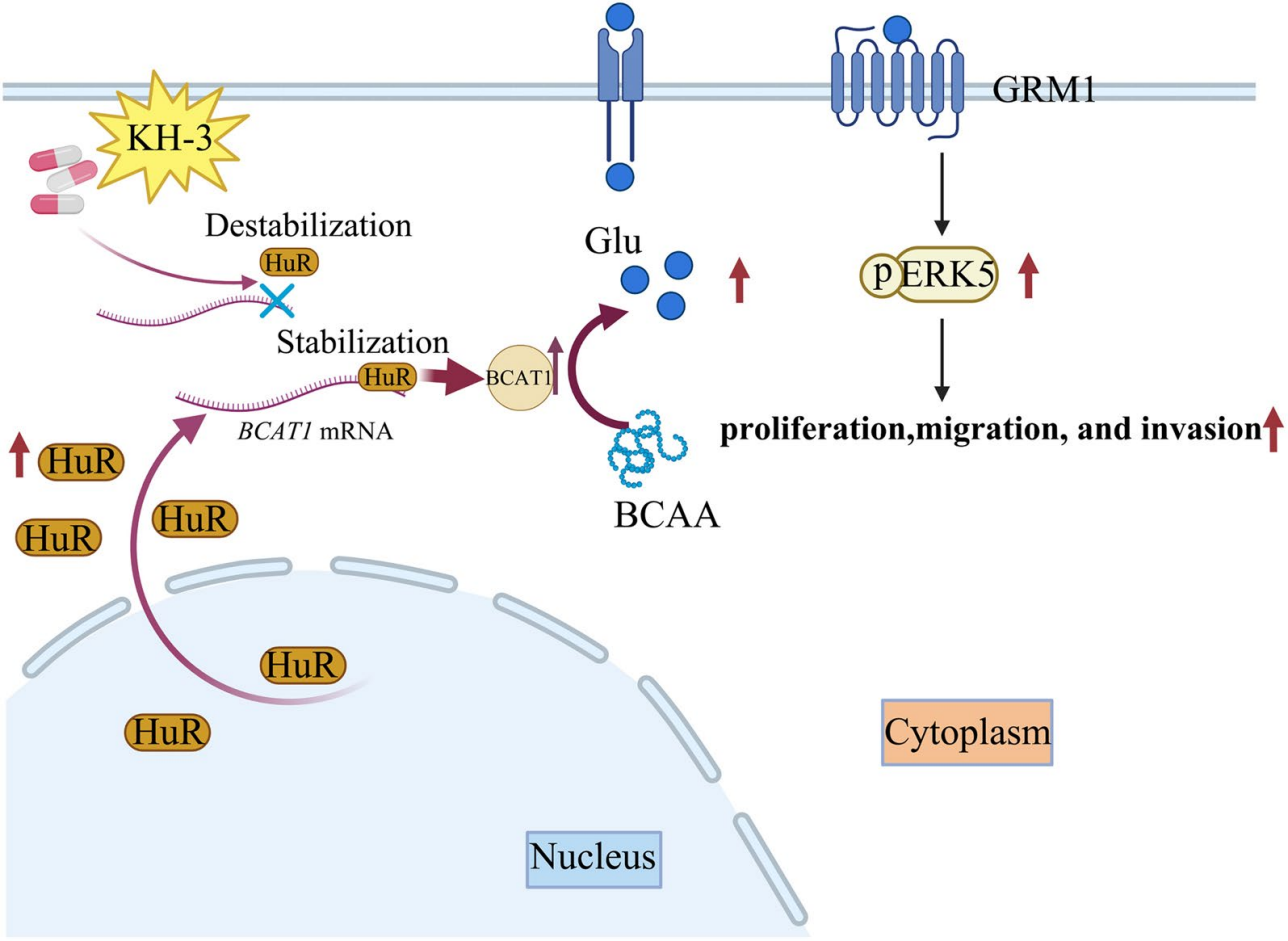

**Supplementary Information:**

The online version contains supplementary material available at 10.1186/s12967-024-04970-w.

## Introduction

Prostate cancer (PCa) is the second most common cancer in men worldwide [1]. Castration-resistant prostate cancer (CRPC) is the late stage of prostate cancer and is insensitive to androgen deprivation therapy (ADT). The survival of patients who progress to CRPC is dramatically decreased [2]. Although novel hormone therapy pharmaceuticals such as abiraterone, enzalutamide, and the poly (ADP-ribose) polymerase inhibitor olaparib improve the prognosis of CRPC, tumours inevitably develop resistance to these treatments [3–5]. Therefore, the identification of novel targets and therapeutics for CRPC patients is urgently needed for the management of CRPC.

In addition to the effects of classic hormones, evidence from genetic, bioinformatics and molecular biological studies suggests that abnormalities in amino acid metabolism play crucial roles at various stages of prostatic carcinogenesis and tumour progression [6–8]. Abnormal amino acid metabolism can promote the development of CRPC [9]. Targeting the amino acid metabolism network in tumour cells inhibits prostate cancer cell growth and increases chemosensitivity [10]. Consequently, an attractive strategy to prevent CRPC progression would be to regulate amino acid metabolism in tumour cells.

Human antigen R (HuR), which is an RNA-binding protein, is a human embryonic lethal abnormal vision-like protein bearing three RNA recognition motifs [11–13]. HuR is known to promote carcinogenesis and treatment resistance in the different cancers, such as breast cancer, colon cancer and glioma, by interacting with a subset of mRNAs related to hormone receptors, inflammation and proliferation [14] because HuR post transcriptionally regulates proliferation, apoptosis, senescence, inflammation and immunity by binding and stabilizing the target gene mRNA [15]. However, HuR was also reported to be upregulated and could play a role in abnormal amino acid metabolism [16]. Considering that cytoplasmic HuR expression is higher in more aggressive CRPC than in hormone-sensitive prostate cancer (HSPC) [17] and that the inhibitor KH-3, which blocks the HuR-mRNA interaction, can significantly mitigate the progression of breast and pancreatic cancer [18, 19], we hypothesized that HuR plays a role in CRPC progression by regulating amino acid metabolism. However, the target of HuR in CRPC progression and the specific molecular mechanism are unknown, and whether inhibiting HuR can alleviate CRPC remains unclear.

In this study, we demonstrated for the first time that branched chain amino-acid transaminase 1 (BCAT1) is a novel target gene of HuR and mediated the effect of HuR on the promotion of CRPC progression. A mechanistic investigation revealed that HuR positively regulated the expression of BCAT1 at the posttranscriptional level. Upregulation of BCAT1 promoted CRPC progression by activating glutamate metabotropic receptor 1 (GRM1)/ERK5 signalling. We also showed that the HuR inhibitor KH-3 effectively suppressed the progression of CRPC by disrupting the interaction between HuR and *BCAT1* mRNA. Our results indicate that the HuR/BCAT1 axis is a promising novel therapeutic target for the treatment of CRPC.

## Materials and methods

### Clinical tissue acquisition

Human hormone-sensitive prostate cancer tissues and CRPC tissues were obtained from prostate biopsies of patients who were treated at Chongqing University Cancer Hospital. Histopathological diagnosis was performed according to the International Society of Urological Pathology criteria and confirmed by an independent pathologist. This study was approved by the Ethics Committee of Chongqing University Cancer Hospital. Written informed consent was obtained from each patient.

### Bioinformatics analysis

*HuR* and *BCAT1* mRNA expression data were extracted from the Gene Expression Omnibus (GEO) (http://www.ncbi.nlm.nih.gov/geo) dataset GSE74367 and used for differential expression analysis. UALCAN (https://ualcan.path.uab.edu/analysis.html) was used to analyse the relationship between HuR expression and the Gleason scores of PCa cases. The expression correlation of HuR and BCAT1 was analysed with Spearman in TCGA (T3bN0, n = 26).

### Cell culture and reagents

The human CRPC cell lines PC3 and DU145, the human HSPC cell line LNCaP, and the human normal prostatic epithelial cell line RWPE-1 were purchased from the American Type Culture Collection (ATCC, USA) and were tested for the absence of mycoplasma contamination. The cells were maintained in RPMI-1640 medium (Gibco, USA) supplemented with 10% foetal bovine serum (Gibco, USA) and antibiotics (100 IU/mL penicillin G). RWPE-1 cells were cultured in keratinocyte growth medium supplemented with human recombinant epidermal growth factor (5 ng/mL) and bovine pituitary extract (0.05 mg/mL) (ScienCell, USA). The cells were cultured at 37 °C in 5% CO_2_. The GRM1 inhibitor JNJ16259685, L-glutamic acid, and actinomycin D were purchased from MedChemExpress (MCE, USA). The HuR inhibitor KH-3 was a kind gift from Prof. Liang Xu [20].

### Construction of stable knockout and overexpression cells

HuR was knocked out in PC3 and DU145 cells by the CRISPR-Cas9 technique. The lentiCRISPRV2 vector was purchased from AddGene, and the control single guide RNA (sgRNA) and HuR sgRNA were cloned and inserted into the vector as previously described [21, 22]. A detailed description is provided in the online supplementary materials and methods.

### RNA sequencing and data analysis

The RNA-Seq dataset contained four groups and compared PC3-sgControl with PC3-KO1 cells and DU145-sgControl with DU145-KO4 cells. RNA-seq was performed on the Illumina HiSeq 2500 platform. Other analyses, including volcano mapping and Gene Ontology (GO) enrichment analysis, were performed using the OmicShare tool, which is a free online platform for data analysis (https://www.omicshare.com/tools). The sequencing data were uploaded to the Gene Expression Omnibus.

### RNA interference

The small interfering RNAs (siRNAs) si-BCAT1-1 (sense: 5′-GAACUGUGUUCACGGAUCATT-3′, antisense: 5′-UGAUCCGUGAACACAGUUCTT-3′) and si-BCAT1-2 (sense: 5′-GAGUCAAGAAGCCUACCAATT-3′, antisense: 5′- UAAGUCCGGAGGAUCUUUCTT-3′) and negative control RNAs (si-NC, sense: 5′-GGCUCUAGAAAAGCCUAUGCTT-3′, antisense: 5′- GCAUAGGCUUUUCUAGAGCCTT-3′) were synthesized by GenePharma Company (China). Transient transfection was performed using Lipofectamine 3000 reagent (Invitrogen, USA) according to the manufacturer’s protocol. The cells were harvested after 24 h for functional analysis.

### Proliferation assay

Cells were seeded at 5 × 10^3^ cells/well in 96-well plates with three duplications and incubated in RPMI-1640 medium with indicated regents for 0, 24, 48 and 72 h. Following incubation with 10 μL of CCK-8 solution for 4 h, the absorbance of each well was measured at OD 450 nm. Cells without gene modification or incubated with vehicle were served as control. In addition, the proliferation of PC3 and DU145 with indicated treatment was measured by a CCK-8 Kit (Beyotime, China).

### Colony formation assay

Cells were seeded on 6-well plates at 500 cells/well in triplicate. After being incubated in complete RPMI-1640 medium supplemented with the indicated reagents for 14 days, the cells were washed with phosphate-buffered saline (PBS) twice, fixed with 4% paraformaldehyde (Biosharp, China) for 10 min and stained with 0.1% crystal violet solution (Solarbio, China) within 10 min for further analysis. Colonies were counted by an independent investigator who was blinded to the experimental design. Cells without gene modification or those that were incubated with the vehicle served as controls.

### Migration and invasion assays

The migration and invasion of PC3 and DU145 cells were analysed using Transwell chambers or Matrigel-coated invasion chambers containing 8 μm pore filters (Corning, USA), respectively. Briefly, cells were seeded at a density of 1 × 10^5^ cells/well in the upper chamber of 24-well plates with or without Matrigel coating. RPMI-1640 medium with vehicle (DMSO), GRM1 inhibitor JNJ16259685 (80 μM) or KH-3 (10 μM) was placed in the upper chamber. Complete medium was placed in the lower chamber as a chemotactic agent. Transwell or invasion assay systems were then incubated at 37 °C with 5% CO_2_ for 24 h. The migration or invasion cells were fixed with 4% paraformaldehyde and stained with crystal violet solution. Non-invasive cells were removed from the upper surface of the membrane by cotton swabs. Cells without gene modification or incubated in medium with vehicle were served as control.

### RNA immunoprecipitation (RIP) assay

The RIP assay was performed with a Magna RIP Kit (Millipore, USA) according to the manufacturer’s instructions. PC3 and DU145 cells were washed with ice-cold PBS and collected by centrifugation into complete RIP lysis buffer on ice for 5 min and followed by immediate freezing at − 80 °C. The protein A/G magnetic beads pre-coated with 4 μg anti-HuR antibodies or mouse IgG (Santa Cruz, USA) were incubated with cell lysates at 4 °C overnight. Beads containing immunoprecipitated RNA–protein complex were then washed three times with cell lysis buffer. Subsequently, co-immunoprecipitated RNA was detected by qRT-PCR analysis.

### Cellular thermal shift assay (CETSA)

CETSA is used to measure the intracellular binding efficiency of a drug to a target protein [23]. PC3 cells were incubated with or without KH-3 (10 μM) for 1 h, after which the cells were collected and suspended in PBS supplemented with a complete protease inhibitor cocktail. The cells were equally divided into 12 tubes (50 μL/tube), and each tube was heated for 3 min at the indicated temperature using a PCR thermocycler followed by cooling at 25 °C for 3 min. Subsequently, the cell suspensions were freeze‒thawed three times with liquid nitrogen and centrifuged at 20,000 ×*g* for 20 min at 4 °C. The soluble fractions were then subjected to western blotting, and the band intensities were measured and normalized to that of β-tubulin.

### Glutamate assay

A total of 2 × 10^6^ vector- and BCAT1-overexpressing PC3 and DU145 cells were harvested and washed in cold PBS. Glutamate concentrations were then measured by a glutamate assay kit (Abcam, US) according to the manufacturer’s instructions. The absorbance of each well was measured at OD = 450 nm. The glutamate concentration of each sample was calculated according to the standard curve.

### Quantitative reverse transcription-PCR (qRT‒PCR) and RNA stability analysis

Total RNA was isolated with TRIzol™ reagent (Invitrogen, USA), and cDNA was synthesized with an iScript cDNA Synthesis Kit (Bio-Rad, USA). RNA expression was analysed by qRT-PCR using iQ SYBR Green Supermix (Bio-Rad, USA). Relative expression was calculated by normalization to GAPDH values via the 2^−△△Ct^ method. The primer sequences are listed in Additional file 1: Table S1. To assess the stability of *BCAT1* mRNA, cells were treated with actinomycin D (5 μg/mL) for the indicated times. RNA was subsequently extracted as described above, and the remaining transcript abundance was determined by qRT-PCR.

### Western blotting

After total protein was extracted by RIPA lysis buffer supplemented with a protease inhibitor cocktail (Roche, USA), the concentration was measured using a BCA Kit (Beyotime, China), and western blotting was performed as previously described [24]. The antibodies used in this study are listed in Additional file 1: Table S2. A detailed description is provided in the online supplementary materials and methods.

### Generation of fluorescently labelled PC3 cells

PC3 cells with or without HuR knockout or BCAT1 overexpression were labelled to determine the expression of firefly luciferase genes using recombinant LentiV-Luci viruses. A detailed description is provided in the supplementary materials and methods online.

### Animal experiments

For a detailed description of the tumour xenografts, tumour imaging, and bone destruction evaluation, please see the online supplementary materials and methods.

### Immunohistochemical staining

Immunohistochemical staining was performed on PCa specimens and mouse tumour xenograft tissues using the avidin–biotin peroxidase complex method. A detailed description is provided in the online supplementary materials and methods.

### Statistical analysis

The data are presented as the mean ± standard error of the mean (SEM). After determining the homogeneity of variance with the Bartlett test, a t test or one-way analysis of variance followed by the Student–Newman–Keuls test was used to evaluate statistical significance among multiple groups. Values of *p* < 0.05 were considered to indicate statistical significance. The experiments were performed in triplicate. The exact sample sizes and number of replicates are stated in the figure legends.

## Results

### HuR deficiency attenuates the malignant progression of CRPC in vitro and in vivo

**Fig. 1:**
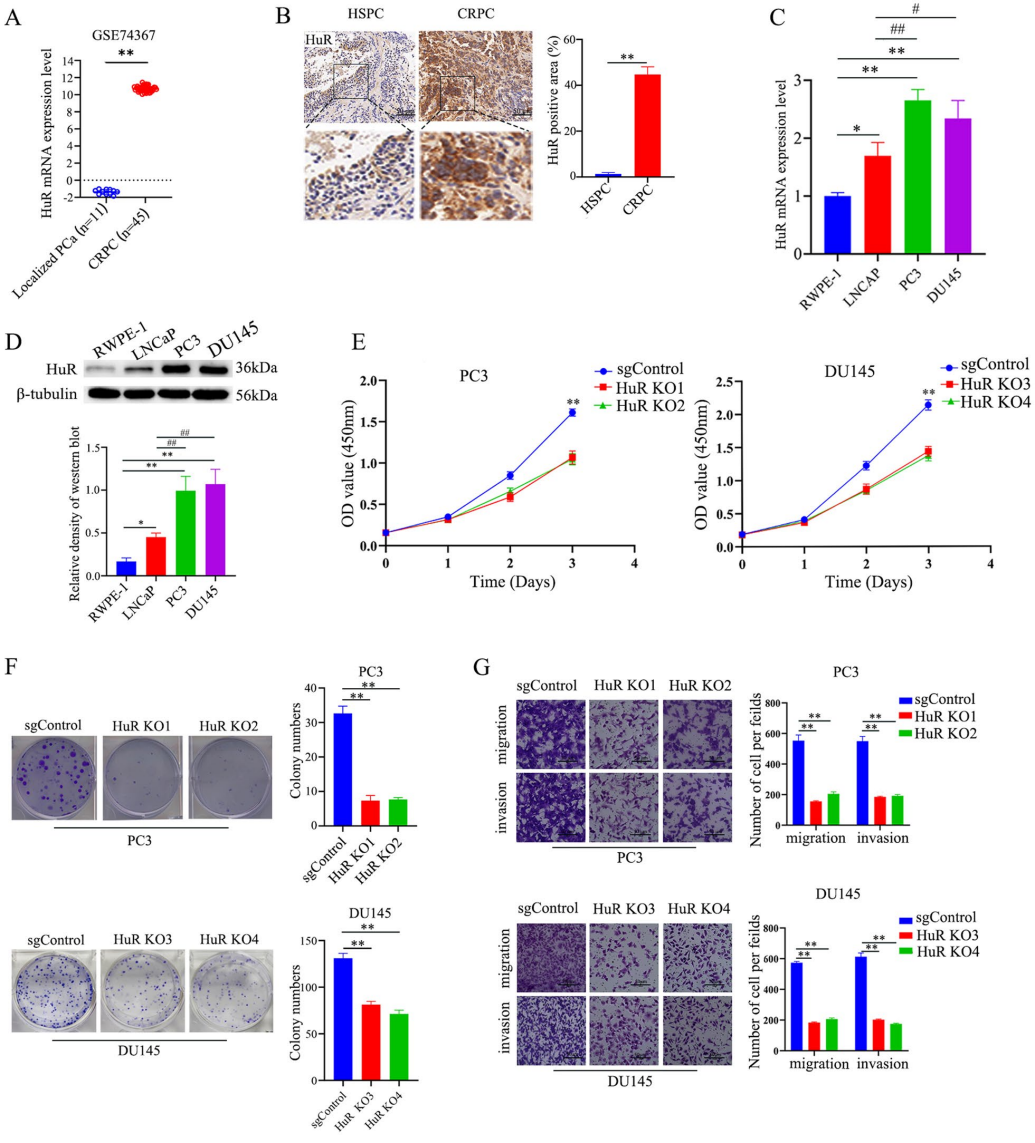
HuR deficiency attenuates the malignant progression of CRPC cells in vitro*.* **A** HuR expression in CRPC tissues compared with LPC tissues in the GSE74367 database. **B** Representative images showing immunostaining (scale bar: 50 μm) of HuR in human HSPC tissues and CRPC tissues. The dashed square outlines the higher magnification image. The right panel shows the percentages of positive areas (brown) of HuR expression in each type of tissue (n = 5). **C** Representative qPCR data showing HuR mRNA expression in the different cell lines.** D** Representative western blots showing HuR expression in the different cell lines. The right panel shows the expression normalized to that of β-tubulin. **E** The proliferation of sgControl and two HuR KO clones of PC3 and DU145 cells, as measured by CCK-8 assays. **F** HuR KO inhibited colony formation in PC3 and DU145 cells (left). The right show the number of clones. **G** Migration and invasion assays of sgControl cells and two HuR KO clones of PC3 and DU145 cells. The right show the corresponding statistical analysis of the number of invasive and migrated cells. *p* values are shown for each comparison (^*^p < 0.05, ^**^p < 0.01, ^#^*p* < 0.05, ^##^*p* < 0.01)

To examine the role of HuR in CRPC progression, we first performed differential expression analysis of the GSE74367 dataset to investigate the association between HuR expression and PCa progression. As shown in Fig. 1A, the mRNA expression of *HuR* was significantly upregulated in CRPC compared with localized prostate cancer (LPC). Immunohistochemical staining showed that HuR expression was significantly upregulated in CRPC tissues compared to HSPC tissues (Fig. 1B). Subsequently, a panel of PCa cell lines and the human normal prostatic epithelial cell line RWPE-1 were used to investigate the basal expression of HuR. The results showed that the mRNA and protein expression levels of HuR in CRPC cells (PC3 and DU145) were significantly higher than those in HSPC cells (LNCap) and normal prostatic epithelial cells (Fig. 1C and D). Data from the TCGA database also revealed that the expression level of HuR was positively correlated with the Gleason score of PCa (Additional file 1: Fig. S1). These results indicate that an increase in HuR is positively correlated with the malignant phenotype of PCa and may participate in prostate cancer progression.

To verify the role of HuR in the malignant behaviors of CRPC cells, we genetically knocked out HuR in PC3 and DU145 cells by the CRISPR-Cas9 technique. We identified eight clones of PC3 cells and four clones of DU145 cells with HuR expression deficiency at both the transcriptional and translational levels after sgRNA transfection (Additional file 1: Fig. S2A and S2B). Two clones of PC3 (KO1 and KO2) and DU145 (KO3 and KO4) cells whose colony formation inhibition was consistent with HuR deficiency were used for subsequent investigations. Growth curve and colony formation assays showed that HuR KO clones grew much slower than sgControl cells (Fig. 1E and F). Furthermore, HuR deficiency inhibited the migration and invasion of PC3 and DU145 cell clones (Fig. 1G). These results indicate that HuR deficiency inhibits the malignant biological behavior of CRPC cells in vitro.

**Fig. 2:**
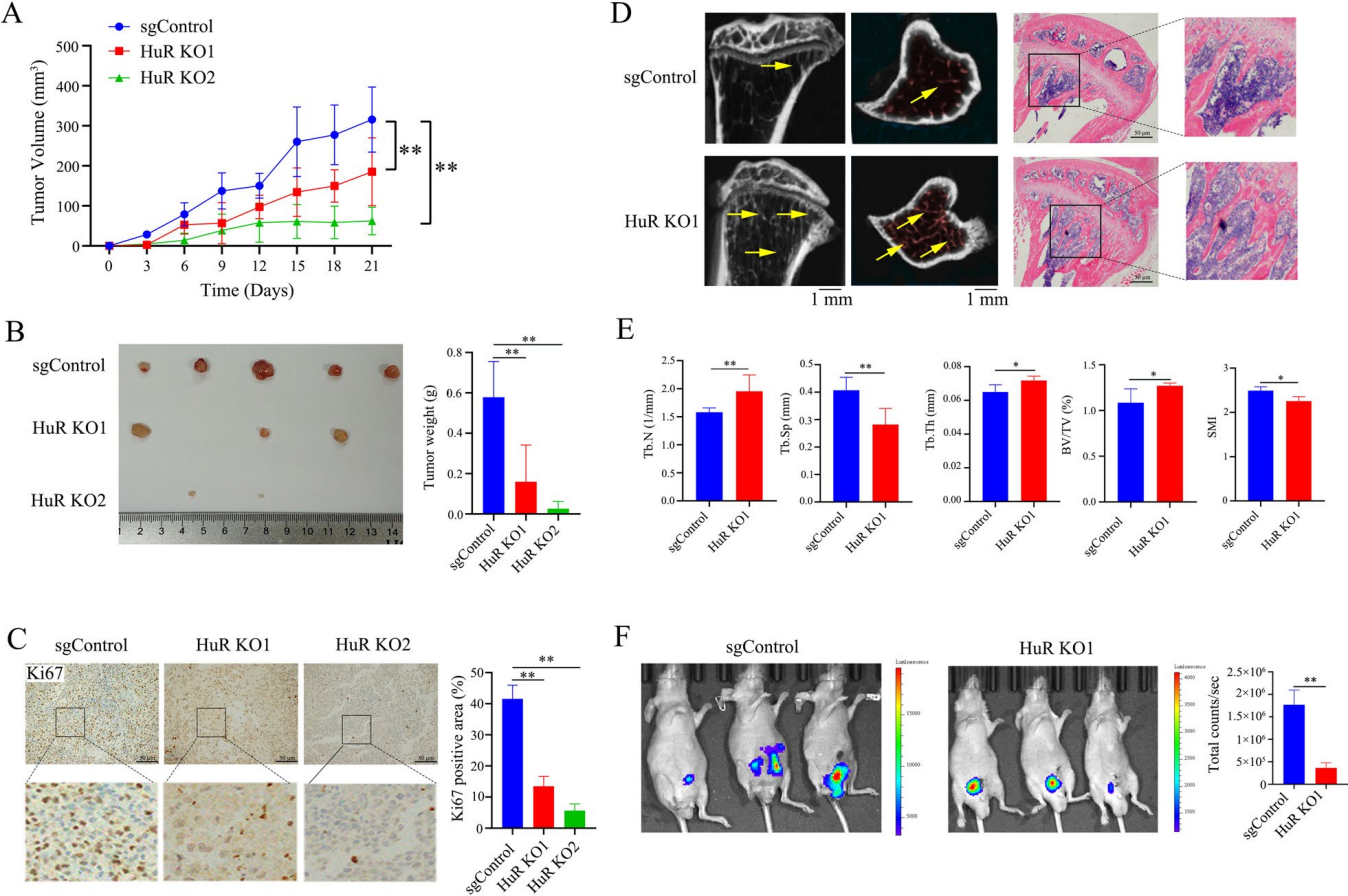
HuR deficiency ameliorates the malignant progression of CRPC in vivo*. A* The tumour xenograft mouse model was established by inoculating nude mice with d sgControl or HuR KO clones of PC3 cells. Tumour growth curves showing the average volume of tumours in each group (n = 5). **B** After 21 days, the mice were sacrificed, and the size (left panel) and weight (right) of the tumour xenografts were measured (n = 5). **C** Representative immunostaining (scale bar: 50 μm) of Ki67 in sgControl and HuR KO tumour xenografts. The dashed square outlines the higher magnification image. The right panel shows the percentages of Ki67-positive areas (brown) in each group of tumour xenografts. **D** Representative micro-CT scan (left) and H&E staining (right) showing increased bone trabeculae and decreased destruction of bone trabeculae in nude mouse tibias inoculated with HuR KO1 PC3 cells compared to those inoculated with sgControl cells. The arrowheads denote bone trabecula. The dashed square outlines the higher magnification image of the bone trabecula (scale bar: 100 μm). **E** Quantitation of the trabecular number (Tb.N), thickness (Tb.Th), separation (Tb.Sp), and percent trabecular area (BV/TV), as well as the structural model index (SMI) in nude mouse tibias inoculated with sgControl or HuR KO1 cells (*p < 0.05, **p < 0.01). **F** In vivo images of tumours showing fewer tumour xenografts and metastatic lesions in nude mice that were orthotopically inoculated with HuR KO1 cells compared to those inoculated with sgControl cells (n = 3). The right panel shows the quantitative fluorescence intensity of tumour lesions in each type of nude mouse. *p* values are shown for each comparison (^*^p < 0.05, ^**^p < 0.01)

To further evaluate the role of HuR in the malignant behavior of CRPC cells in vivo, we inoculated nude mice with sgControl or two PC3 HuR KO clones. The xenograft model revealed that knocking out HuR substantially inhibited tumour size and weight compared to the effects on xenograft tumours generated from sgControl PC3 cells (Fig. 2A and B). Immunostaining showed that the expression of Ki67 in xenograft tumours generated from HuR KO1 and HuR KO2 cells was significantly reduced compared with that in xenograft tumours generated from sgControl PC3 cells, indicating that the proliferation of PC3 cells was inhibited by HuR deficiency in vivo (Fig. 2C).

Bone is the most common site of CRPC metastasis, and bone metastasis is one of the leading causes of mortality in patients with advanced prostate cancer [25]. To clarify the role of HuR in PCa bone metastasis, PC3 (sgControl and HuR KO1) cells were inoculated into the tibias of male nude mice. Micro-CT and H&E staining of the tibias revealed that bone destruction was significantly alleviated in the tibias of mice injected with HuR KO1 cells compared to that in the tibias of mice injected with sgControl cells (Fig. 2D). Increases in trabecular numbers, thickness, and the percent trabecular area and decreases in trabecular separation and structural model indices were observed in the HuR KO1 group (Fig. 2E). Moreover, in the fluorescent orthotopic tumour xenograft model, the sizes of localized tumour xenografts and metastatic lesions were significantly reduced in mice that were inoculated with HuR KO1 cells compared to those in mice that were inoculated with sgControl cells (Fig. 2F). These results suggest that HuR deficiency alleviates the malignant progression of CRPC cells in vivo.

### BCAT1 is a potential target of HuR in CRPC cells

**Fig. 3:**
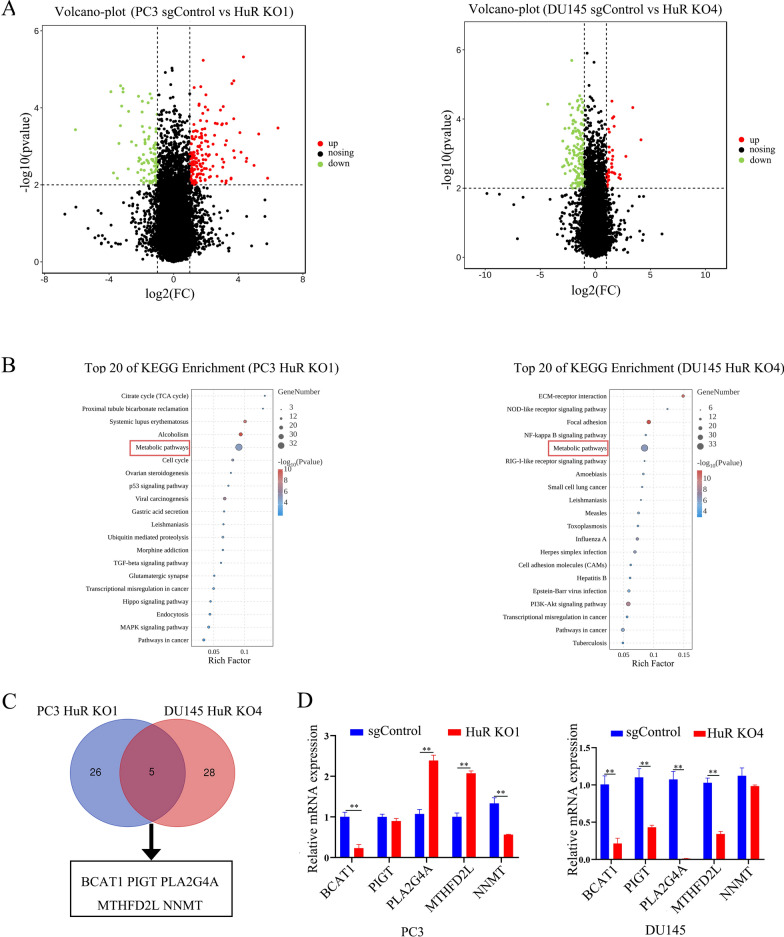
BCAT1 is a potential target of HuR in CRPC cells.** A** Volcano dot plots showing upregulated genes (red dots) and downregulated genes (green dots) in HuR KO1 PC3 and HuR KO4 DU145 cells compared to sgControl cells. **B** Enrichment analysis of the KEGG pathways showed that the differentially expressed genes (DEGs) in PC3 and DU145 cells with HuR KO were enriched in metabolic pathways. **C** Venn diagram showing the number of targets and intersecting genes (BCAT1, PIGT, PLA2G4A, MTHFD2L, and NNMT) identified among the DEGs in the metabolic pathway by RNA-seq in both PC3 and DU145 cells. **D** Relative mRNA levels of BCAT1, PIGT, PLA2G4A, MTHFD2L, and NNMT in PC3 and DU145 cells with HuR KO were measured by qRT-PCR. p values are shown for each comparison (**p < 0.01)

HuR promotes tumour progression by binding to the mRNA 3′-UTR of target genes and promoting translation [12, 26]. However, the target gene by which HuR promotes CRPC progression is unknown. To identify the target genes of HuR in the malignant progression of CRPC, RNA-seq was performed to compare the transcript changes between sgControl and HuR KO clones of PC3 and DU145 cells. Compared with sgControl clones of PC3 and DU145 cells, 888 and 887 downregulated genes were identified in PC3 HuR KO1 and DU145 HuR KO4 cells, respectively, based on fold changes in expression (≤ − 1) (Fig. 3A). Kyoto Encyclopaedia of Genes and Genomes (KEGG) analysis revealed metabolic pathways were present in the top 20 enriched KEGG pathways among downregulated genes in both HuR-deficient PC3 and DU145 cells (Fig. 3B). The Venn diagram showed that five overlapping differentially expressed genes (*BCAT1*, *PIGT*, *PLA2G4A*, *MTHFD2L,* and *NNMT*) in the metabolic pathway in HuR-deficient PC3 and DU145 cells. Furthermore, the qRT-PCR results showed that the expression of BCAT1, which is a branched-chain amino acid transaminase, was significantly downregulated in both HuR-deficient PC3 and DU145 cells compared with control cells (Fig. 3C and D). These results indicate that BCAT1 is a potential target gene of HuR in CRPC cells.

### HuR positively regulates BCAT1 expression by stabilizing *BCAT1* mRNA in CRPC cells

**Fig. 4:**
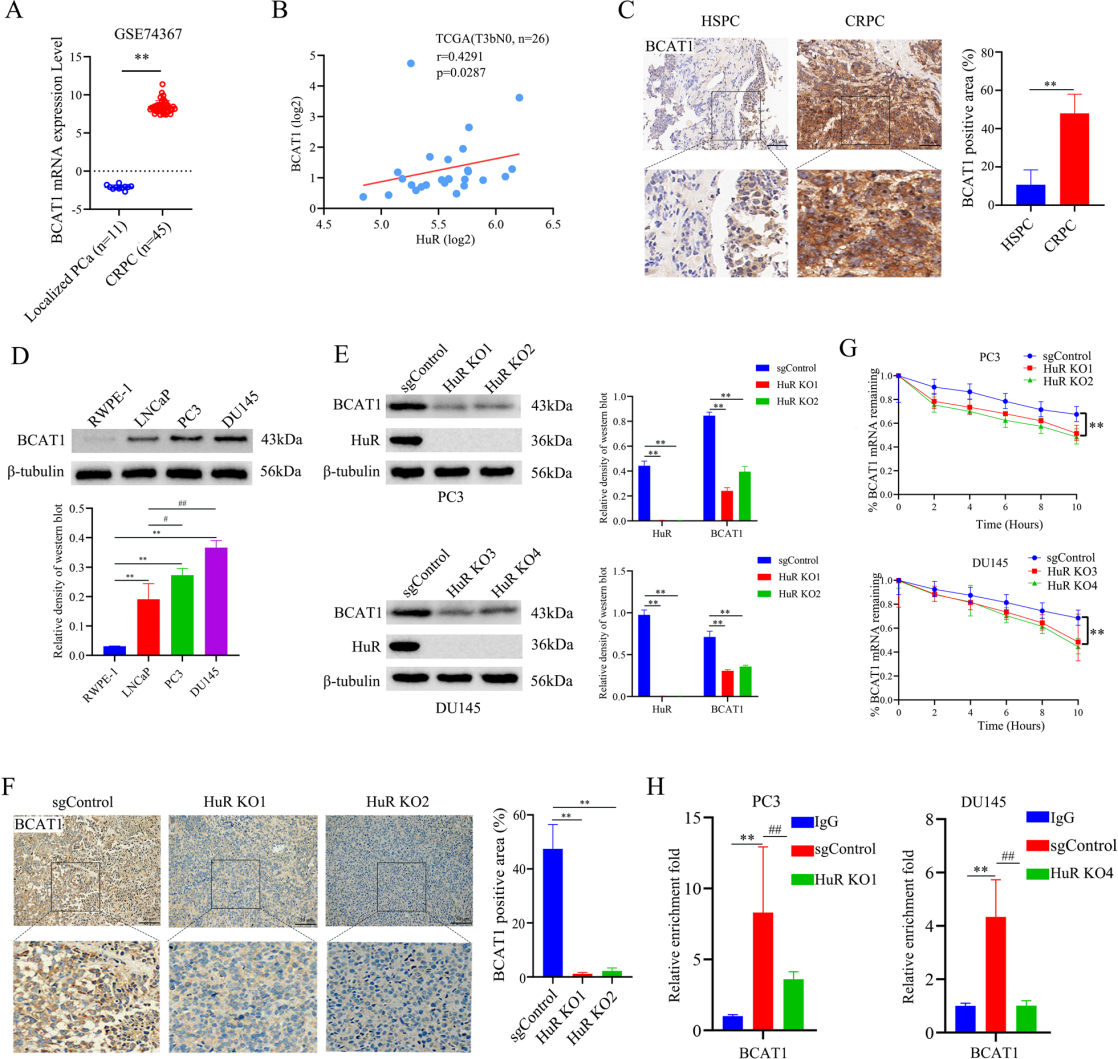
HuR positively regulates BCAT1 expression by stabilizing *BCAT1* mRNA in CRPC cells. **A** The expression of BCAT1 in CRPC tissues compared with LPC tissues in the GSE74367 database. **B** The correlation between the expression levels of HuR and BCAT1 in the TCGA database was analysed with Spearman correlation (T3bN0, n = 26). **C** Representative immunostaining (scale bar: 50 μm) of BCAT1 in human HSPC tissues and CRPC tissues. The dashed square outlines the higher magnification image. The right panel shows the percentages of BCAT1-positive areas (brown) in each type of tissue (n = 5). **D** Representative western blots showing BCAT1 expression in the different cell lines. The lower show the expression intensity normalized to that of β-tubulin. **E** Representative western blots showing BCAT1 and HuR expression in HuR KO clones of PC3 and DU145 cells. The lower show the expression intensity normalized to that of β-tubulin. **F** Representative images (scale bar: 50 μm) of BCAT1 expression in tumour xenografts generated from sgControl or HuR KO PC3 cells. The dashed square outlines the higher magnification image. The right panel shows the percentages of BCAT1-positive areas (brown) in each group of xenografts (n = 5). **G** Treatment with 5 μg/mL actinomycin D reduced the half-life of *BCAT1* mRNA in HuR KO clones of PC3 and DU145 cells. **H** RIP qRT-PCR analysis showed that the binding of HuR to *BCAT1* mRNA was affected by HuR knockout in PC3 and DU145 cells. Isotype IgG was used as a negative control for the HuR antibody. *p* values are shown for each comparison (^**^p < 0.01, ^#^*p* < 0.05, ^##^*p* < 0.01)

An increase in BCAT1 expression has been reported in several cancers and is closely associated with malignant progression [24, 27–29], but its expression in prostate cancer is unknown. Analysis of the GSE74367 dataset revealed that *BCAT1* mRNA expression was significantly upregulated in CRPC tissues compared with LPC tissues (Fig. 4A). To determine whether HuR regulates BCAT1, we examined the relationship between HuR and BCAT1. As shown in Fig. 4B, *BCAT1* mRNA level in PCa samples in public datasets (TCGA) were positively correlated with *HuR* mRNA expression (P = 0.0287, R = 0.4291). Immunohistochemical staining also revealed that BCAT1 expression was significantly upregulated in CRPC tissues compared to HSPC tissues (Fig. 4C). The expression levels of BCAT1 in PCa cell lines were compared, and the results showed that BCAT1 was markedly increased in the CRPC cell lines PC3 and DU145 (Fig. 4D). To determine whether BCAT1 is regulated by HuR, we examined BCAT1 expression in HuR KO clones. As shown in Fig. 4E, there was an obvious reduction in BCAT1 protein levels in HuR KO clones compared to sgControl clones among PC3 and DU145 cells. Moreover, immunostaining revealed that the expression of BCAT1 in tumour xenografts derived from HuR KO cells was decreased compared with that in tumour xenografts derived from sgControl cells (Fig. 4F).

HuR typically stabilizes its target mRNAs and promotes translation [26]. To determine whether the stability of *BCAT1* mRNA is affected by HuR, cells were treated with actinomycin D, and *BCAT1* mRNA levels were measured by qRT-PCR to determine the half-life of the BCAT1 transcript. As shown in Fig. 4G, the half-life of *BCAT1* mRNA in HuR KO cells was significantly shorter than that in sgControl cells. To further assess whether BCAT1 is a direct target gene of HuR, we performed RIP followed by qRT-PCR on PC3 and DU145 cells. As expected, compared to that in the IgG controls, the marked enrichment of *BCAT1* mRNA in HuR-immunoprecipitated samples confirmed the binding of HuR to *BCAT1* mRNA. However, the enrichment of *BCAT1* mRNA was dramatically reduced in the HuR KO clones (Fig. 4H). Taken together, these data suggest that HuR interacts with *BCAT1* mRNA and upregulates its expression at least in part by stabilizing *BCAT1* mRNA.

### BCAT1 acts as an oncogene in CRPC

**Fig. 5:**
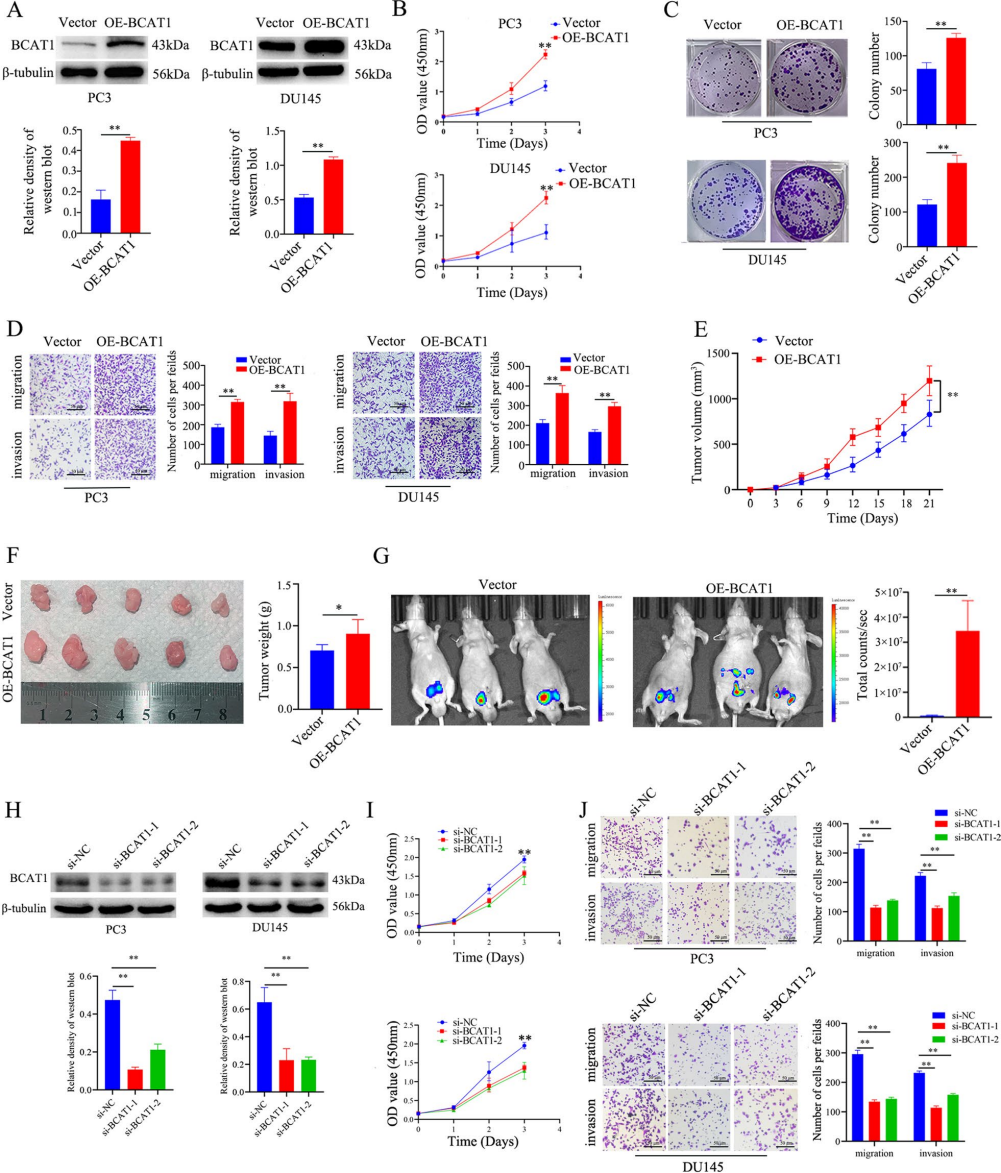
BCAT1 acts as an oncogene in CRPC. **A** BCAT1 protein expression levels in Lenti-vector- or Lenti-BCAT1-infected PC3 and DU145 cells. The lower show the expression intensity normalized to that of β-tubulin. **B** The proliferation of Lenti-vector- or Lenti-BCAT1-infected PC3 and DU145 cells was measured by a CCK-8 assay. **C** Lenti-BCAT1 infection increased colony formation in PC3 and DU145 cells. The right show the number of clones. **D** The migration and invasion of Lenti-vector- and Lenti-BCAT1-infected PC3 and DU145 cells were determined by transwell assays. The right show the quantitation of invasive and migrated cells. **E** The volume of tumour xenografts in nude mice inoculated with Lenti-BCAT1-infected PC3 cells compared to that of mice inoculated with vector-treated PC3 cells at 21 days (n = 5). **F** Size (left) and weight (right) of tumour xenografts in nude mice inoculated with Lenti-vector- or Lenti-BCAT1-infected PC3 cells for 21 days (n = 5). **G** In vivo images of tumours showing increased tumour xenografts and metastatic lesions in nude mice that were orthotopically inoculated with Lenti-BCAT1-infected PC3 cells at 21 days (n = 3). The right panel shows the fluorescence intensity of tumour lesions in each type of nude mouse. **H** The protein expression levels of BCAT1 in PC3 and DU145 cells transfected with si-NC, si-BCAT1-1 or si-BCAT1-2. The lower show the expression intensity normalized to that of β-tubulin. **I** The proliferation of si-NC-infected, si-BCAT1-1-infected, and si-BCAT1-2-infected PC3 and DU145 cells was measured by a CCK-8 assay. **J** The migration and invasion of si-NC, si-BCAT1-1, and si-BCAT1-2 infected PC3 and DU145 cells were determined by transwell assays. The right show the quantitation of invasive or migrated cells. *p* values are shown for each comparison (^*^p < 0.05, ^**^p < 0.01)

Since BCAT1 is directly regulated by HuR, we next evaluated the role of BCAT1 in CRPC progression. We first overexpressed BCAT1 in PC3 and DU145 cells by lentiviral infection (Fig. 5A). Subsequently, several assays were carried out to determine changes in cell behavior. As shown in Fig. 5B–D, BCAT1 overexpression significantly increased the proliferation, colony formation, migration, and invasion of PC3 and DU145 cells. In addition, the volume and weight of the tumour xenografts derived from BCAT1-overexpressing PC3 cells were markedly increased compared to those of tumour xenografts derived from sgControl PC3 cells (Fig. 5E, F). Similarly, the fluorescent orthotopic tumour xenograft model showed that the sizes of localized tumour xenografts and metastatic lesions were notably increased in mice inoculated with BCAT1-overexpressing PC3 cells compared with mice inoculated with vector control cells at 3 weeks (Fig. 5G). However, knockdown of BCAT1 with siRNA inhibited the proliferation, migration, and invasion of PC3 and DU145 cells (Fig. 5H–J). These findings suggest that BCAT1, which is a target gene of HuR, acts as an oncogene in CRPC.

### BCAT1 mediates the oncogenic effect of HuR on CRPC cells

**Fig. 6:**
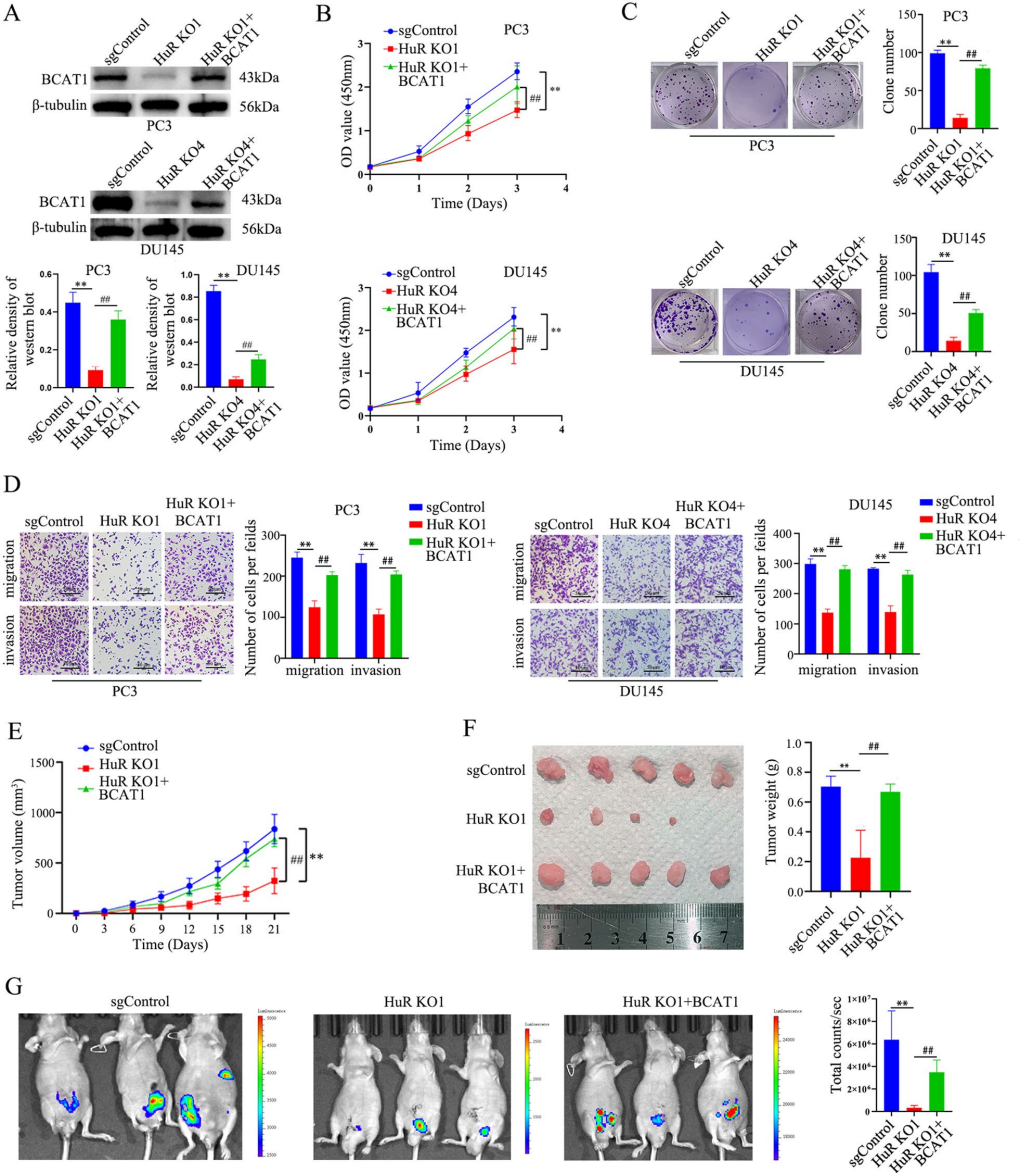
BCAT1 mediates the oncogenic effect of HuR in CRPC cells. **A** The protein expression levels of BCAT1 in HuR KO PC3 and DU145 cells with or without BCAT1 rescue were measured by western blotting. The lower show the expression intensity normalized to that of β-tubulin. **B** The proliferation of HuR KO cells with or without BCAT1 rescue was measured by a CCK-8 assay. **C** Clonogenic ability was determined in HuR KO cells with or without BCAT1 rescue. The right show the number of clones. **D** The migration and invasion of HuR KO cells with BCAT1 rescue were determined by transwell assays. The right show the quantitation of invasive and migrated cells. **E** The volume of tumour xenografts in nude mice inoculated with HuR KO1 PC3 cells harbouring rescued BCAT1 expression compared with that of mice inoculated with vehicle control HuR KO1 cells (n = 5). **F** The size (left) and weight (right) of tumour xenografts were measured on the 21st day after inoculation (n = 5). **G** In vivo images showing increased tumour xenografts and metastatic lesions in nude mice that were orthotopically inoculated with PC3 HuR KO1 cells bearing rescued BCAT1 expression compared to those inoculated with vehicle control HuR KO1 cells at 21 days (n = 3). The right panel shows the quantitative fluorescence intensity of tumour lesions in each type of nude mouse. sgControl cells served as a negative control. *p* values are shown for each comparison (^**^p < 0.01, ^##^*p* < 0.01)

Whether BCAT1, which is a potential target gene of HuR, mediates the carcinogenic effect of HuR on CRPC cells remains to be explored. Therefore, we overexpressed BCAT1 in HuR KO clones of PC3 and DU145 cells (Fig. 6A). The inhibition of CRPC cell proliferation, colony formation, migration, and invasion induced by HuR deficiency was reversed by BCAT1 overexpression (Fig. 6B–D). In addition, a tumour xenograft model was generated in nude mice by subcutaneously injecting the indicated cells to verify whether these in vitro findings were relevant to PCa tumour growth in vivo. Consistent results were obtained from the tumour xenograft model, and BCAT1 overexpression in HuR KO1 cells significantly accelerated the growth of the xenograft tumours compared to the growth of the xenograft tumours derived from HuR KO1 cells without BCAT1 overexpression (Fig. 6E). The size and weight of the tumour xenografts derived from HuR KO1 cells overexpressing BCAT1 were significantly increased compared with those of tumour xenografts derived from HuR KO1 cells without BCAT1 at three weeks (Fig. 6F). The fluorescent orthotopic tumour xenograft model revealed that BCAT1 overexpression in HuR KO1 cells markedly increased the size of the xenograft tumours and metastatic lesions compared to those in mice inoculated with HuR KO1 cells without BCAT1 overexpression (Fig. 6G). Our data suggest that BCAT1 largely mediates the effect of HuR on CRPC.

### The BCAT1-induced aggressive behaviors of CRPC cells are dependent on the activation of ERK5 signalling

**Fig. 7:**
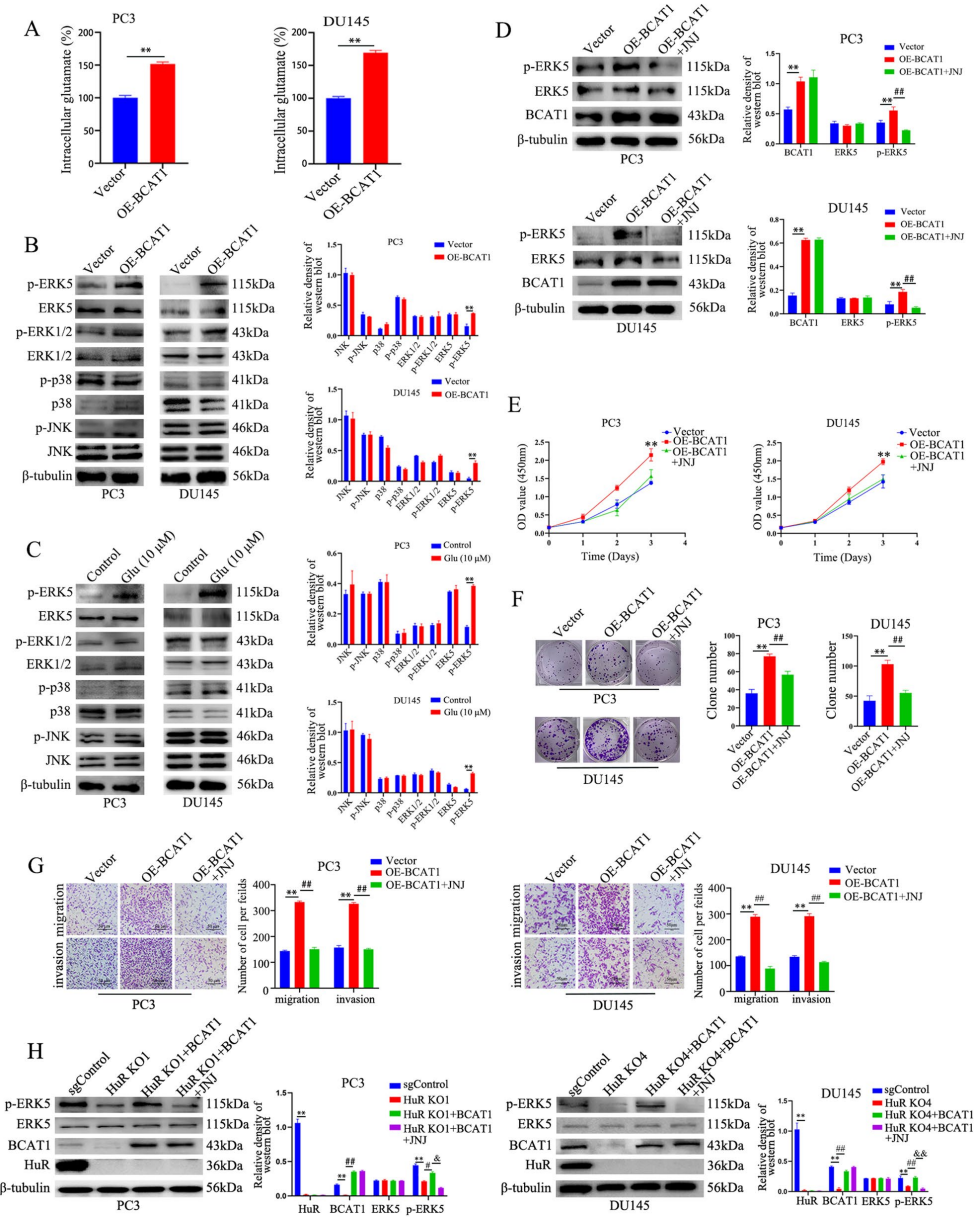
BCAT1-induced aggressive behaviors of CRPC cells are dependent on the activation of ERK5 signalling. **A** Intracellular glutamate levels were determined in BCAT1-overexpressing PC3 and DU145 cells. **B** The protein expression levels of ERK5, p-ERK5, ERK1/2, p-ERK1/2, p38, p-p38, JNK, and p-JNK in BCAT1-overexpressing PC3 and DU145 cells were determined by western blotting. The expression intensity was normalized to that of β-tubulin (right). **C** PC3 and DU145 cells were treated with 10 μM glutamate for 24 h, after which the protein expression levels were analysed by western blotting. The expression intensity was normalized to that of β-tubulin (right panel). **D** BCAT1-overexpressing cells were treated with JNJ16259685 (80 μM) for 24 h, after which the protein expression levels were analysed by western blotting. The expression intensity was normalized to that of β-tubulin (right panel). **E–G** BCAT1-overexpressing cells were treated with JNJ16259685 (80 μM) for 24 h, after which proliferation (**E**), colony formation (**F**), migration, and invasion (**G**) were measured. The right show the quantitative results. **H** Representative immunoblots of HuR, BCAT1, ERK5, and p-ERK5 in HuR KO1 PC3 and HuR KO4 DU145 cells with or without BCAT1 overexpression and incubation with JNJ16259685 (80 μM) for 24 h (left). The right show the expression intensity normalized to that of β-tubulin. *p* values are shown for each comparison (^**^p < 0.01, ^#^*p* < 0.05, ^##^*p* < 0.01, ^&^*p* < 0.05, ^&&^*p* < 0.01)

Previous studies have confirmed that BCAT1 is the key enzyme that catalyses branched-chain amino acid metabolism and produces different branched-chain keto acids and glutamate [30]. Glutamate levels directly correlate with the Gleason score and aggressiveness of PCa, and inhibiting glutamate release or blocking GRM1 decreases the growth, migration, and invasion of PCa cells [31]. Therefore, we overexpressed BCAT1 in PC3 and DU145 cells and examined intracellular glutamate concentrations and major downstream signalling pathways of GRM1. The results showed that BCAT1 overexpression significantly increased the intracellular concentration of glutamate (Fig. 7A). Previous studies have shown that GRM1 is mostly related to the activation of the MAPK signalling pathway [32, 33], and we examined the expression of key proteins in the MAPK signalling pathway. As shown in Fig. 7B, BCAT1 overexpression in PC3 and DU145 cells did not significantly affect the total protein or phosphorylation levels of ERK1/2, p38, or JNK but did increase the phosphorylation level of ERK5. We also observed similar results when PC3 and DU145 cells were treated with glutamate (10 μM) for 24 h (Fig. 7C). These results indicated that BCAT1 overexpression activated the GRM1/ERK5 signalling pathway in CRPC cells. To further confirm that BCAT1 activated the ERK5 signalling pathway, cells overexpressing BCAT1 were treated with the GRM1-specific inhibitor JNJ16259685 (80 μM). We found that incubation with JNJ16259685 not only abrogated the phosphorylation of ERK5 in PC3 and DU145 cells overexpressing BCAT1 (Fig. 7D) but also inhibited the BCAT1 overexpression-induced proliferation, colony formation, migration, and invasion of PC3 and DU145 cells (Fig. 7E–G). Moreover, the GRM1 inhibitor JNJ16259685 abrogated the effect of BCAT1 overexpression on the phosphorylation of ERK5 in HuR-deficient PC3 and DU145 cells (Fig. 7H). These results suggest that HuR controls the malignant behaviors of CRPC cells via BCAT1/ERK5 signalling.

### KH-3 inhibits BCAT1 expression and attenuates CRPC cell progression in vitro and in vivo

**Fig. 8:**
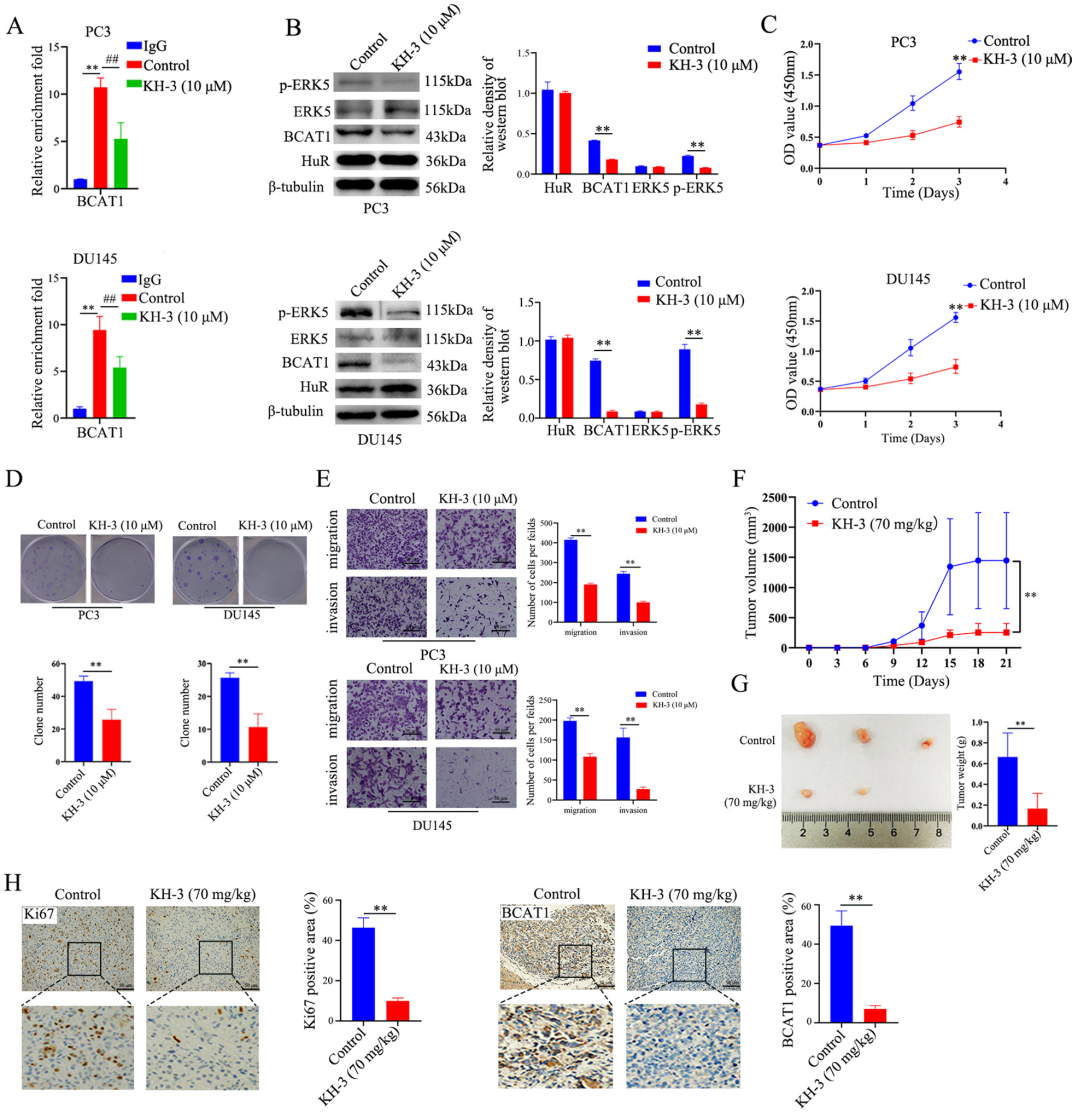
KH-3 inhibits BCAT1 expression and attenuates CRPC cell progression in vitro and in vivo*.* **A** RIP qRT-PCR analysis showed that the binding of HuR to *BCAT1* mRNA was affected by KH-3 (10 μM) in PC3 and DU145 cells. Isotype IgG was used as a negative control for the HuR antibody. **B** Representative immunoblots showing ERK5, p-ERK5, HuR, and BCAT1 expression in PC3 and DU145 cells incubated with KH-3 (10 μM) for 24 h (left). The right show the expression intensity normalized to that of β-tubulin. **C**–**E** PC3 and DU145 cells were treated with KH-3 (10 μM) as above, after which proliferation (**C**), colony formation (**D**), migration, and invasion (**E**) were measured. The right show the quantitative results. **F** The volume of tumour xenografts in nude mice inoculated with PC3 cells an intraperitoneally injected with 70 mg/kg KH-3 twice per week starting from the first day after tumours grew to an approximate diameter of 4 mm and continuing to the third week (n = 3). **G** After 21 days, the mice were sacrificed, and the size (left panel) and weight (right panel) of the tumour xenografts in nude mice were measured (n = 3). **H** Representative immunostaining (scale bar: 50 μm) of Ki67 and BCAT1 expression in tumour xenografts in nude mice inoculated with PC3 cells and treated with KH-3 at 21 days. The dashed square outlines the higher magnification image. The right show the percentages of Ki67- and BCAT1-positive areas (brown) (n = 3). *p* values are shown for each comparison (^**^p < 0.01, ^##^*p* < 0.01)

A previous study demonstrated that KH-3 is a HuR-mRNA interaction inhibitor that could suppress the growth of breast cancer [18] and pancreatic cancer [19]. To further confirm whether KH-3 can bind to endogenous HuR and disrupt the interaction between HuR and *BCAT1* mRNA in PCa, we performed CETSA and RIP assays. Compared with the DMSO control, KH-3 (10 μM) treatment induced thermal stabilization of HuR, suggesting that KH-3 binds directly to HuR in PC3 cells (Additional file 1: Fig. S3). The immunoprecipitation results showed that *BCAT1* mRNA was significantly enriched by the HuR antibody compared to IgG, confirming that BCAT1 mRNA was a direct target of HuR in PC3 and DU145 cells. KH-3 clearly disrupted the interaction between HuR and *BCAT1* mRNA (Fig. 8A).

We therefore investigated the effects of KH-3 on the BCAT1/ERK5 pathway and on the biological characteristics of CRPC cells. As shown in Fig. 8B, treatment of PC3 and DU145 cells with KH-3 in vitro markedly decreased BCAT1 expression and the phosphorylation of ERK5. Furthermore, proliferation, colony formation, migration, and invasion were inhibited after KH-3 treatment, similar to the effect of HuR deficiency (Fig. 8C–E). To assess the therapeutic effects of KH-3, we examined its cytotoxicity against prostate cancer cell lines and human normal prostatic epithelial cells. KH-3 was highly cytotoxic to PC3 and DU145 cells with an IC_50_ less than 10 µM, while the cytotoxicity of KH-3 against RWPE-1 cells was much lower, as indicated by an IC_50_ greater than 33.7 µM (Additional file 1: Fig. S4). Nude mice inoculated with PC3 cells were treated with 70 mg/kg KH-3 twice per week beginning on the first day after the tumours had grown to approximately 4 mm in diameter. The results showed that KH-3 significantly slowed tumour growth and reduced the volume and size of xenograft tumours (Fig. 8F, G). In addition, immunohistochemical staining confirmed that the administration of KH-3 reduced Ki67 and BCAT1 expression in tumour xenografts derived from PC3 cells (Fig. 8H). These studies demonstrated that functional inhibition of HuR by KH-3 attenuated BCAT1 expression and suppressed CRPC progression in vitro and in vivo, similar to the effects observed after HuR knockout.

## Discussion

Despite the previous success of ADT and new antiandrogen therapy, most patients with CRPC ultimately develop resistance to this treatment [3, 4], and patient prognosis dramatically deteriorates [2]. To determine the molecular mechanism of CRPC occurrence, various pharmaceuticals have been developed to fight this disease. However, none of them completely eradicates this disease. Docetaxel, which is a tubulin inhibitor, has been proven to be effective in treating CRPC; however, chemoresistance and adverse side effects limit further survival of CRPC patients [34]. Novel androgen synthesis and androgen receptor inhibitors, such as abiraterone and enzalutamide, significantly improve the prognosis of CRPC patients, but androgen receptor mutations limit their long-term efficacy [3, 4]. Poly (ADP-ribose) polymerase inhibitors, such as olaparib and niraparib, have been approved for use in CRPC treatment by the FDA and EMA, but these agents are limited to CRPC patients bearing mutations in genes involved in homologous recombination repair [5, 35]. Hence, there is a great unmet medical need for CRPC treatment due to a lack of effective and nonselective therapeutic targets.

Although previous studies shown that HuR plays a carcinogenic role in a variety of tumours [14, 36], the function and underlying mechanisms of HuR in CRPC are unclear. In this study, we demonstrated that knocking out HuR in CRPC cell lines not only inhibited cell proliferation, migration, and invasion in vitro but also attenuated the malignant progression of PC3 cells in vivo*.* These results suggest that HuR may serve as a promising therapeutic target for inhibiting CRPC progression. Mechanistically, RNA-seq and bioinformatics analyses of HuR KO cells indicated that *BCAT1* mRNA was a target of HuR. Immunoblotting and staining verified that overexpression of HuR was followed by the upregulation of BCAT1 expression in CRPC cell lines and clinical CRPC tissues, and genetic modification further confirmed that HuR knockout decreased BCAT1 expression in CRPC cells and tumour xenografts. Furthermore, knockout of HuR significantly shortened the half-life of BCAT1 in CRPC cells, suggesting that BCAT1 lost stability and was quickly decomposed in the absence of HuR. RIP assays proved that the HuR antibody could enrich* BCAT1* mRNA, while knockout of HuR reduced the precipitation of *BCAT1* mRNA. These data confirm that BCAT1 is a direct target gene of HuR. Moreover, functional inhibition of HuR with KH-3 could disrupt the interaction between HuR and *BCAT1* mRNA, thereby reducing the expression of BCAT1. One of the mechanisms by which HuR promotes tumour progression is by binding to the 3′-UTR of an oncogene and promoting its expression. Therefore, the positive regulation of BCAT1 by HuR likely results from the binding of HuR to the 3′-UTR of *BCAT1* mRNA. However, the specific binding sites of HuR in the BCAT1 3′-UTR need further study.

Alterations in gene expression that drive metabolic reprogramming are key features of cancer cells in response to increased stress and energy requirements. These alterations help cancer cells proliferate and survive in unfavourable environments [37–39]. BCAT1 is an enzyme that transfers the amine group on branched-chain amino acids to alpha-ketoglutarate, thereby producing glutamate [30]. Since the role of BCAT1 overexpression in glioma development was reported in 2013 [40], BCAT1 has received increasing attention as a prognostic tumour marker and an attractive target for cancer therapy in many types of tumours [27]. Studies have indicated that BCAT1 overexpression is positively correlated with cancer progression and poor outcomes [27]. Inhibiting BCAT1 in a human primary glioblastoma cell line produced smaller tumours in mice [41]. Similarly, subcutaneous transplantation of BCAT1-deficient non-small lung cancer (NSCLC) cells into mice resulted in impaired xenograft tumour formation [42]. In this study, we showed for the first time that BCAT1 was a novel factor driving CRPC by affecting proliferation, colony formation, migration, and invasion in PC3 and DU145 cells. Moreover, the carcinogenic effect of BCAT1 on CRPC was further confirmed by tumour xenografts derived from PC3 cells overexpressing BCAT1. We also found that BCAT1 overexpression ameliorated the inhibition of malignant activity in HuR-knockout CRPC cells. These results are consistent with the finding that BCAT1 overexpression induces drug resistance, proliferation, migration, and invasion in cancer cells [42–44]. Overall, the HuR-BCAT1 axis is an important mechanism driving CRPC progression, and the effects of HuR on CRPC are at least partially dependent on BCAT1.

Previous studies confirmed that BCAT1 is involved in many pathways to regulate downstream factors during tumour progression [45]. BCAT1 promotes cell proliferation by controlling the expression of the cell cycle inhibitor p27Kip1 and is required for hormone-independent breast tumour proliferation; silencing of BCAT1 leads to a massive reduction in tumour volume in an orthotopic triple-negative xenograft model [46]. In this study, we demonstrated that BCAT1 overexpression increased the concentration of glutamate, the ligand for GRM1, and activated ERK5 phosphorylation through GRM1. Treatment with the GRM1 inhibitor JNJ16259685 resulted in the dephosphorylation of ERK5 and a decrease in the proliferation, colony formation and invasion of BCAT1-overexpressing PC3 cells, indicating that the role of BCAT1 in promoting CRPC cell progression depended on glutamate-induced GRM1/ERK5 signalling activation. In addition, knockout of HuR resulted in the downregulation of BCAT1 and phosphorylation of ERK5, while BCAT1 overexpression rescued the phosphorylation of ERK5 in HuR-deficient cells, suggesting that ERK5 is regulated by the HuR-BCAT1 axis. Considering that BCAT1 overexpression increased glutamate levels and glutamate-induced GRM1/ERK5 signalling activation, so glutamate is the link connecting the HuR-BCAT1 axis, metabolic effects and ERK5. Furthermore, the protumorigenic activity of HuR was mediated by BCAT1, and BCAT1-induced CRPC cell progression was dependent on glutamate-induced GRM1/ERK5 signalling activation. Thus, these results demonstrate that the protumorigenic activity of HuR on CRPC cells is dependent on BCAT1-induced and glutamate-mediated ERK5 metabolic signalling. Theoretically, when BCAT1 catalyses the catabolism of BCAAs to produce glutamate, the production of branched-chain keto acids will also increase. However, the role of branched-chain keto acids catalysed by BCAT1 in CRPC was not examined in this study and needs to be studied further.

As a novel synthetic small molecule inhibitor of HuR, KH-3 has been shown to suppress the progression of breast cancer and pancreatic cancer [18, 19]. However, the anticancer effect of KH-3 on PCa and the underlying mechanism are still unclear. In this study, we revealed for the first time that KH-3 did not affect HuR expression but inhibited the enrichment of *BCAT1* mRNA by interfering with HuR-mRNA interactions. Moreover, our investigation clearly showed the therapeutic potential of KH-3 in CRPC in vitro and in vivo, which is closely related to a decrease in BCAT1 expression and ERK5 activation, which is consistent with the idea that suppressing BCAT1 can inhibit tumour development [42–44]. However, our experiments did not validate the dose-dependent effects of KH-3 on tumorigenicity in preclinical xenograft models, which requires further testing. A recent study showed that KH-3 and docetaxel combination therapy synergistically inhibited cell proliferation and tumour growth in breast cancer [47], but the effect of this combination on CRPC needs to be further evaluated.

In summary, our study is the first to identify HuR as a potential tumour promoter that accelerates CRPC progression by post-transcriptionally upregulating BCAT1 and activating ERK5 signalling in CRPC cells. HuR deficiency or pharmacologically targeting HuR is a promising approach for attenuating CRPC progression by suppressing BCAT1 expression and subsequent ERK5 signalling, indicating that the HuR/BCAT1/ERK5 axis plays a critical role in the progression of CRPC and may serve as a potential novel target for CRPC therapy.

### Supplementary Information

**Additional file 1: Figure S1.** HuR is positive correlated with the malignant progression of CRPC. Expression level of HuR in prostate cancer tissues base on Gleason scores searched in TCGA database. **Figure S2.** Knockout HuR by CRISPR-Cas9 in PC3 and DU145. **A** Knockout HuR in PC3 and DU145 cells by CRISPR-Cas9 technique and the expression of HuR in the clones of PC3 and DU145 cells was determined by qRT-PCR. **B** Western blots analyzed the protein levels of HuR in eight clones of PC3 and four clones of DU145 cells. The two clones numbered 2 and 25 in PC3 are defined as HuR KO1 and HuR KO2. The two clones numbered 3 and 8 in PC3 are defined as HuR KO3 and HuR KO4. **Figure S3.** The intracellular binding efficiency of KH3 to HuR. **A** PC3 cells incubated with or without KH-3 (10 μM) for 1 h were subjected to CETSA assay. The expression of HuR proteins were detected at different temperatures. β-tubulin was used as an internal control. **B** CETSA curves of the relative band intensity at the indicated temperature was calculated based on the band intensity at 42 °C. Representative western blot results from one experiment. **Figure S4.** Dose–response curves with IC_50_ values for KH-3. **A** The IC_50_ values for RWPE-1 cells generated from MTT assay following exposure to KH-3 for 24 h. **B** The IC_50_ values for PC3 cells generated from MTT assay following exposure to KH-3 for 24 h. **C** The IC_50_ values for DU145 cells generated from MTT assay following exposure to KH-3 for 24 h. **Table S1.** Primer list for qPCR. **Table S2.** Antibody used in this study.

## Data Availability

RNA-seq data are deposited at Gene Expression Omnibus (Accession number: GSE254851). Other data generated or analysed during this study are included in this published article and its supplementary information files or available from the corresponding author upon reasonable request.

## Abbreviations

CRPC: Castration resistant prostate cancer
HuR: Human antigen R
BCAT1: Branched-chain amino transferases 1
PCa: Prostate cancer
ADT: Androgen deprivation therapy
PARP: Poly (ADP-ribose) polymerase 1
HSPC: Hormone sensitive prostate cancer
BCAA: Branched chain amino acids
GRM1: Glutamate metabotropic receptor 1
ERK5: Extracellular signal regulated kinase 5
