# Supplementary material for: HuR promotes castration-resistant prostate cancer progression by altering ERK5 activation via posttranscriptional regulation of BCAT1
Source: J Transl Med. 2024 Feb 18;22:178. doi: 10.1186/s12967-024-04970-w (PMC10874581; doi:10.1186/s12967-024-04970-w)
Supplement: Supplementary file 1 — Additional file 1: Figure S1. HuR is positive correlated with the malignant progression of CRPC. Expression level of HuR in prostate cancer tissues base on Gleason scores searched in TCGA database. Figure S2. Knockout HuR by CRISPR-Cas9 in PC3 and DU145. A Knockout HuR in PC3 and DU145 cells by CRISPR-Cas9 technique and the expression of HuR in the clones of PC3 and DU145 cells was determined by qRT-PCR. B Western blots analyzed the protein levels of HuR in eight clones of PC3 and four clones of DU145 cells. The two clones numbered 2 and 25 in PC3 are defined as HuR KO1 and HuR KO2. The two clones numbered 3 and 8 in PC3 are defined as HuR KO3 and HuR KO4. Figure S3. The intracellular binding efficiency of KH3 to HuR. A PC3 cells incubated with or without KH-3 (10 μM) for 1 h were subjected to CETSA assay. The expression of HuR proteins were detected at different temperatures. β-tubulin was used as an internal control. B CETSA curves of the relative band intensity at the indicated temperature was calculated based on the band intensity at 42 °C. Representative western blot results from one experiment. Figure S4. Dose–response curves with IC50 values for KH-3. A The IC50 values for RWPE-1 cells generated from MTT assay following exposure to KH-3 for 24 h. B The IC50 values for PC3 cells generated from MTT assay following exposure to KH-3 for 24 h. C The IC50 values for DU145 cells generated from MTT assay following exposure to KH-3 for 24 h. Table S1. Primer list for qPCR. Table S2. Antibody used in this study. [file 12967_2024_4970_MOESM1_ESM.docx]

**Additional file 1**

**Materials and methods**

**Construction of stable knockout and overexpressed cells**

The HuR lentiviral sgRNA or control sgRNA was co-transfected into 293T cells with the packaging plasmids pMD2.G and psPAX2 (AddGene, USA) [1,2]. Lentiviral particles were collected to infect PC3 and DU145 and then cells were selected with 2 µg/mL puromycin (Biosharp, China) to obtain stable cell lines. Single clones were generated by limiting dilution. Knockout of HuR expression was verified by qRT-PCR and western blotting. BCAT1 overexpression were performed in naïve and HuR knockout PC3 and DU145 cells by lentiviral transduction. Recombinant lentiviral vectors carrying human *BCAT1* gene were constructed by Siwega Biotechnology Co., Ltd (Siwega, China). The detailed description appears in the online supplementary materials and methods.

**Western blotting**

Total protein (20 μg) from each sample was electrophoresed on 12% sodium dodecyl sulfate–polyacrylamide gel electrophoresis gels. After being transferred to polyvinylidene fluoride (Pierce, USA), protein samples were incubated with the primary antibodies at 4 °C overnight. Antibodies used in this study were listed in Supplementary Table S2. Blots were incubated with the appropriate horseradish peroxidase-conjugated secondary antibodies (Abcam, US) and the membranes were developed with SuperSignal™ chemiluminescence reagent (Pierce, USA) according to the manufacturer’s protocol. Protein expression levels were normalized against β-Tubulin. Band optical density was quantified using NIH Image J.

**Generation of fluorescence labeled PC3 cells**

To construct recombinant LentiV carrying exogenous genes, the firefly luciferase genes were cloned from plasmid pGL3-Basic (Promega, USA) with primer pairs Luc-F/R, respectively. The fragments were provided with a translation initiation (Kozak) sequence and flanked on either side with an Age I restriction site. The PCR products were purified with a gel extraction kit and cloned into the Lentivirus vector, using the Age I site. The resulting plasmids were named LentiV-Luci vector. The LentiV-Luci plasmids were used with virus packaging helper plasmid Helper 1.0 and Helper 2.0 to generate LentiV-Luci virus. After detection of the obtained LentiV-Luci viruses, PC3 cells and PC3 cells with HuR knockout and BCAT1 overexpression were infected with LentiV-Luci (MOI=120) for tumor imaging experiments *in* *vivo*.

**Tumor xenografts**

A suspension of PC3 cells with or without HuR knockout and BCAT1 overexpression (5 × 10^5^ cells in PBS 1:1 mixed with Matrigel) was subcutaneously injected into the right flank of 6-week-old male nude mice from the Center of Experimental Animals (Army Medical University, China). To test the effect of HuR-mRNA interaction inhibitor on tumor growth, KH-3 was intraperitoneal injected to the mice inoculated with vehicle control PC3 cells at 70 mg/kg twice a week from the first day after tumors grew to an approximate diameter of 4 mm. The formation and growth of implanted tumors were measured in volumes every 3 days and monitored for 3 weeks. The mice were then sacrificed, and the xenograft tumors were dissected and subjected to immunohistochemistry staining. The experimental protocols were approved by the Animal Care and Use Committee of Army Medical University.

***In vivo* tumor imaging**

The fluorescent orthotopic tumor xenografts were generated by injection of fluorescent PC3 cells with or without HuR knockout and BCAT1 overexpression into the prostate of 6-week-old male nude mice from the Center of Experimental Animals (Army Medical University, China). Mice bearing orthotopic tumor xenografts received anesthesia with 1.5% isoflurane and were analyzed by in *vivo* tumor imaging using an *in vivo* fluorescence imaging system (PerkinElmer, USA) after 21 days of inoculation. The experimental protocols were approved by the Animal Care and Use Committee of Army Medical University.

**Bone destruction evaluation**

Each 1×10^6^ of PC3 cells with or without HuR knockout were first suspended in 20 μL Hank's balanced salt solution. After general anaesthesia induced by 3% isoflurane inhalation and maintained by 2% isoflurane inhalation, 1×10^6^ of cells were injected into the tibia plateau of 6-week-old male nude mice (Army Medical University, China). The mice were sacrificed by placing them into a CO_2_ box for euthanasia five weeks later. The tibias were collected to receive micro-CT scan (Materialise, Belgium) and haematoxylin-eosin (H&E) staining. The bone lesions were evaluated by the trabecular numbers (Tb.N), thickness (Tb.Th), separation (Tb.Sp) and percent trabecular area (BV /TV=Tb.N×Tb.Th/10) as well as structure model index (SMI) which were calculated by the micro-CT scan carried software (Materialise, Belgium). The experimental protocols were approved by the Animal Care and Use Committee of Army Medical University.

**Immunohistochemistry staining**

After deparaffinization and rehydration through graded solutions of ethanol/water, antigen sites in sections were retrieved by boiling in 0.1M citrate buffer (pH 6.0) for 10 min. Slides were then treated with 3% hydrogen peroxide to inactivate endogenous peroxidase. Following incubation in phosphate-buffered saline containing 10% species-appropriate normal serum for 1h at room temperature to block non-specific binding, sections were incubated with primary antibodies, overnight at 4 °C in a humidified chamber using isotype-matched IgG as negative controls. The bound antibodies were detected using biotinylated secondary antibodies and incubated with HRP-Streptavidin (Abcam, US) at 37 °C for 20 min. After washing, specific expression was visualized using a yellow diaminobenzidine reagent kit (Abcam, US) according to the manufacturer’s instructions, and specimens were counterstained with hematoxylin. For quantitative comparison, the ratios of positively stained areas to total traced areas were determined and expressed as percentage on high-power (×200) images using colour segmentation in Image-Pro Plus by an independent pathologist blinded to the experimental design.

**Cell cytotoxicity assay**

The cells were seeded in 96-well culture plates (5,000 cells/well) and treated with serially diluted test compounds in triplicate. After 24 h, the medium was removed, MTT reagent (Beyotime, China) was added to each well, and the plates were incubated at 37 °C for 1 h. The absorbance was measured with a plate reader at 450 nm. The IC_50_, which is the concentration that causes 50% growth inhibition, was calculated by sigmoid curve fitting using GraphPad Prism 8.0.

**References**

1. Sanjana NE, Shalem O, Zhang F. Improved vectors and genome-wide libraries for CRISPR screening. Nat Methods. 2014;11(8):783-4.

2. Shalem O, Sanjana NE, Hartenian E, Shi X, Scott DA, Mikkelson T, et al. Genome-scale CRISPR-Cas9 knockout screening in human cells. Science. 2014;343(6166):84-7.

**Figures and tables**

**
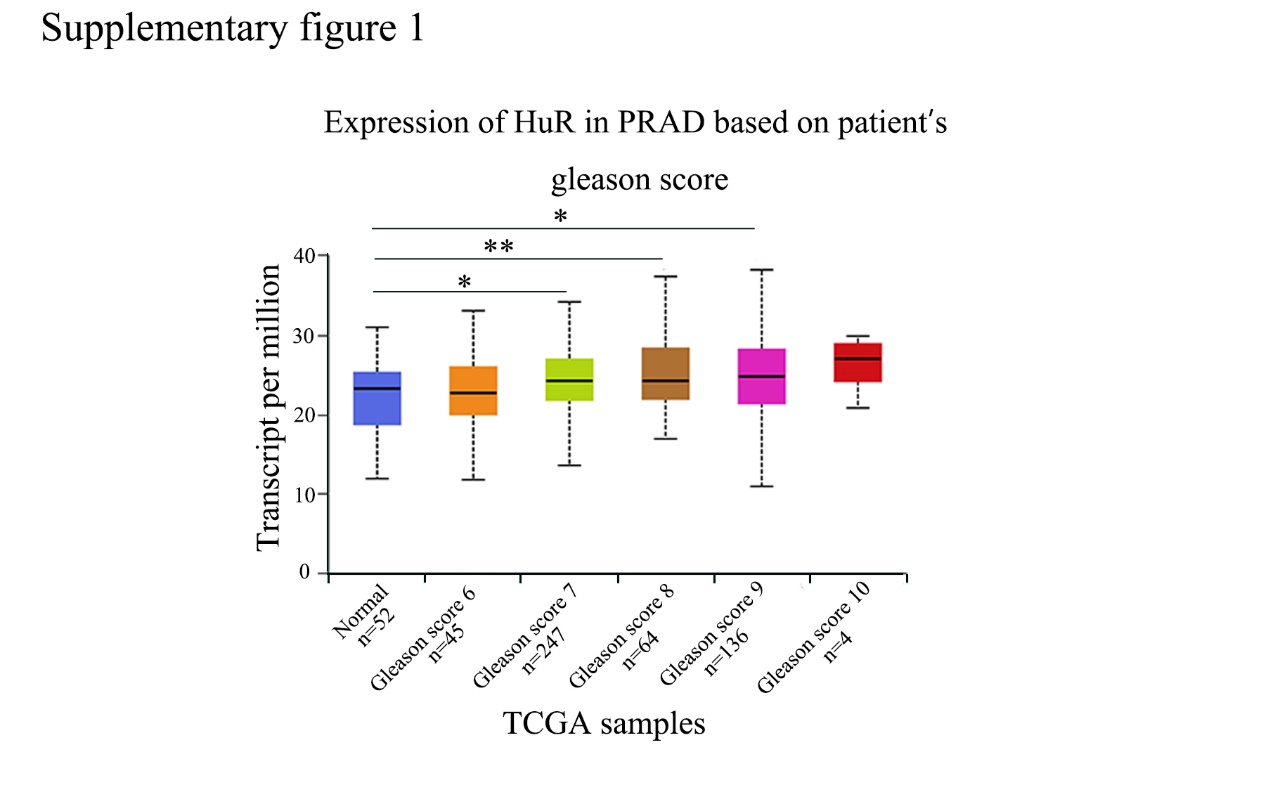
**

**Fig. S1 HuR is positive correlated with the malignant progression of CRPC**

Expression level of HuR in prostate cancer tissues base on Gleason scores searched in TCGA database.


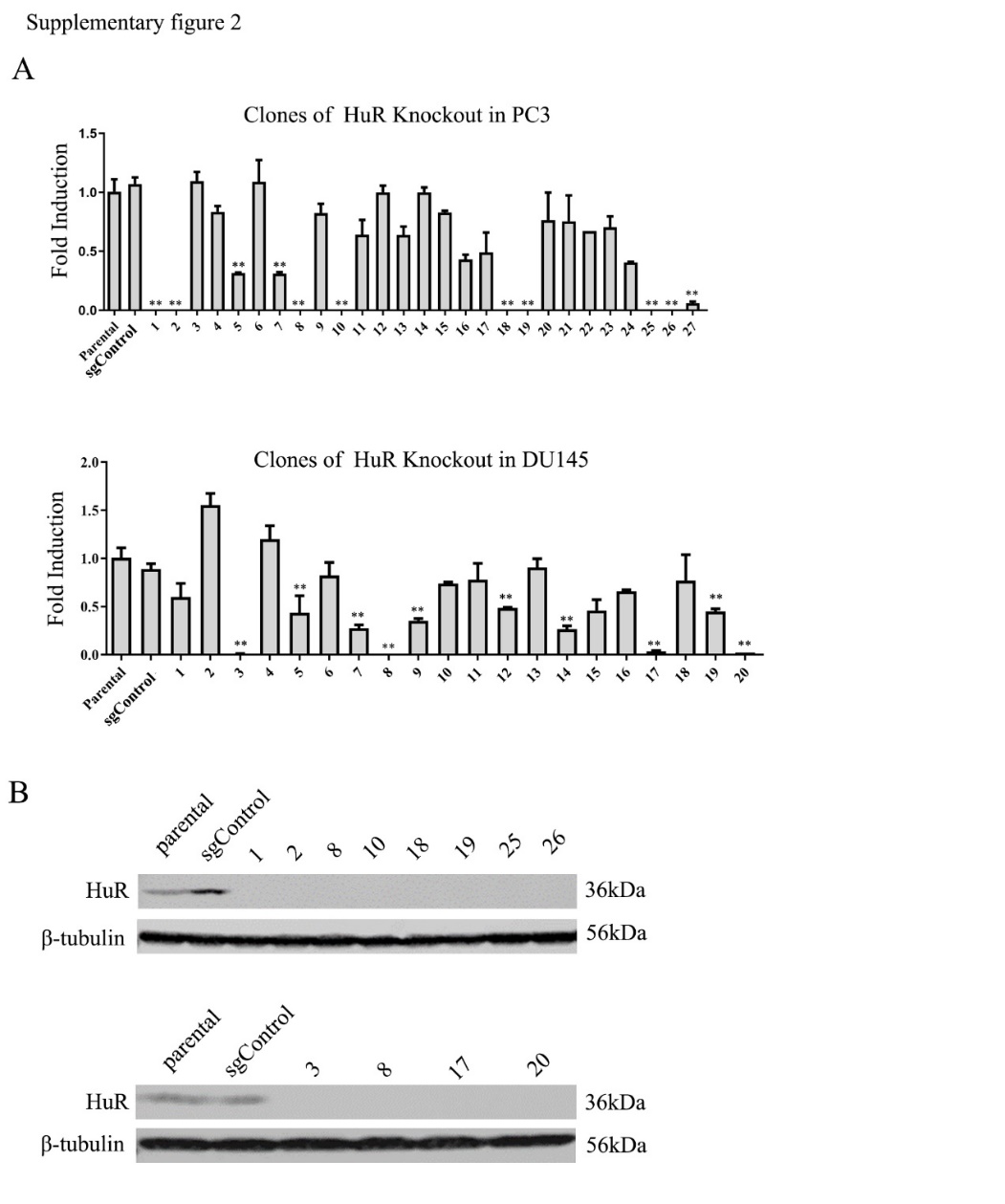


**Fig. S2** **Knockout HuR by CRISPR-Cas9 in PC3 and DU145**

(A) Knockout HuR in PC3 and DU145 cells by CRISPR-Cas9 technique and the expression of HuR in the clones of PC3 and DU145 cells was determined by qRT-PCR. (B) Western blots analyzed the protein levels of HuR in eight clones of PC3 and four clones of DU145 cells. The two clones numbered 2 and 25 in PC3 are defined as HuR KO1 and HuR KO2. The two clones numbered 3 and 8 in PC3 are defined as HuR KO3 and HuR KO4.


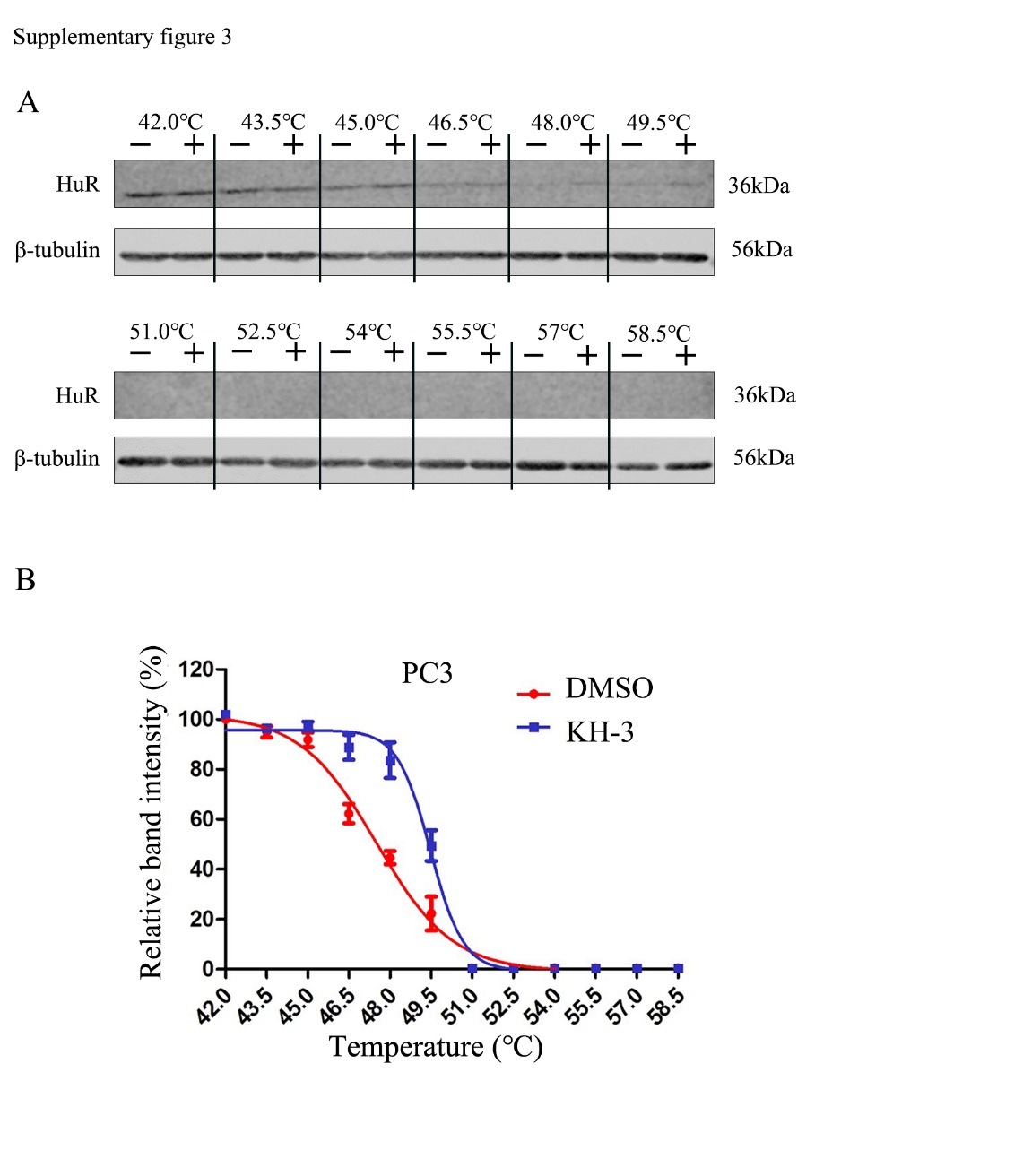


**Fig. S3** **The intracellular binding efficiency of KH3 to HuR**

**(A)** PC3 cells incubated with or without KH-3 (10 μM) for 1 h were subjected to CETSA assay. The expression of HuR proteins were detected at different temperatures. β-tubulin was used as an internal control. **(B)** CETSA curves of the relative band intensity at the indicated temperature was calculated based on the band intensity at 42 °C. Representative western blot results from one experiment.


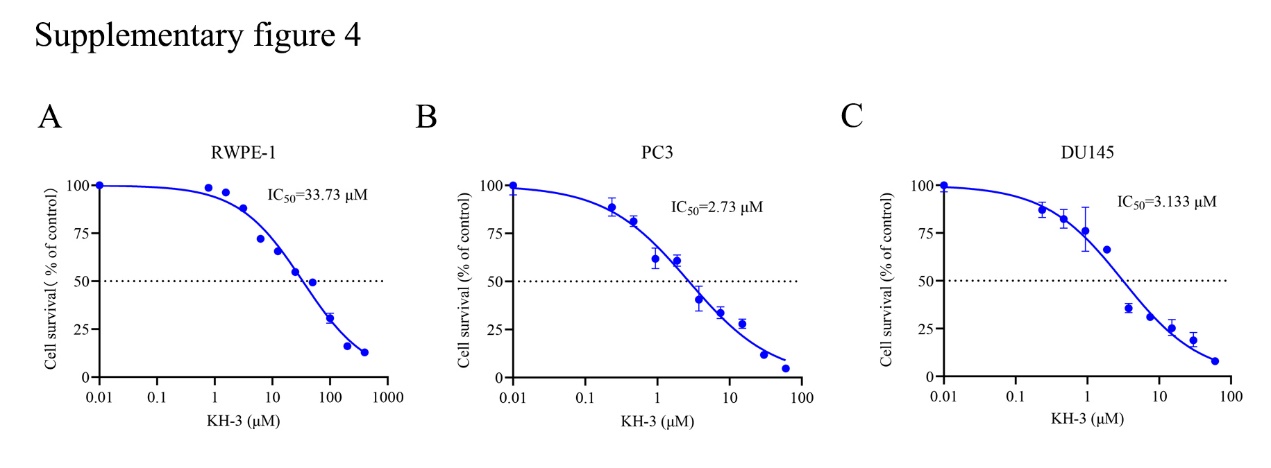


**Fig. S4 Dose-response curves with IC_50_ values for KH-3**

(A)The IC_50_ values for RWPE-1 cells generated from MTT assay following exposure to KH-3 for 24 h. (B) The IC_50_ values for PC3 cells generated from MTT assay following exposure to KH-3 for 24 h.(C) The IC_50_ values for DU145 cells generated from MTT assay following exposure to KH-3 for 24 h.

**Table S1. Primer list for qPCR**

| **Targets** | **Forward primers (5'-3')** | **Reverse primers (5'-3')** |
| --- | --- | --- |
| **GAPDH** | CAGGAGGCATTGCTGATGAT | GAAGGCTGGGGCTCATTT |
| **BCAT1** | TGTCTCAAGGTTTCTGGTCA | GGTGTGGTTTCTGGTCTTTC |
| **NNMT** | TCAGCCAACATTCCTTAGC | GCACCATCATTCTTCTCGT |
| **PIGT** | AAGGACGAGAGAAAAGCAC | CACACAAAGGGGAAGACC |
| **MTHFD2L** | CCAGAAGGAACAGTGGTTTGGC | ACTGTCCTCTGGGCTTGGTGTT |
| **PLA2G4A** | CAGAAAAGTGGGCTAAAATGA | CAGAACAAAGTGGATGATGG |
| **HuR** | ACCAGGCATTGAAAAGACA | AGGAAGGCTGCGAAAAG |

**Table S2. Antibody used in this study**

| **Source** | **Primary antibodies** | **Catalog no.** | **Working dilution** |
| --- | --- | --- | --- |
| **Santa Cruz** | Mouse anti-HuR | sc-5261 | WB,1:1000 IHC,1:500 IP,5μg |
| **Proteintech** | Rabbit anti-BCAT1 | 13640-1-AP | WB,1:1000 IHC,1:500 |
| **Proteintech** | Rabbit anti-ki67 | 28074-1-AP | IHC,1:500 |
| **ZEN-BIOSCIENCE** | Rabbit anti-JNK | R22866 | WB,1:1000 |
| **ZEN-BIOSCIENCE** | Rabbit anti-Phospho-JNK | 381100 | WB,1:1000 |
| **ZEN-BIOSCIENCE** | Rabbit anti-p38 | R25239 | WB,1:1000 |
| **ZEN-BIOSCIENCE** | Rabbit anti-Phospho-p38 | 310091 | WB,1:1000 |
| **ZEN-BIOSCIENCE** | Mouse anti-beta Tubulin | T200608 | WB,1:5000 |
| **Abcam** | Rabbit anti-ERK1/2 | ab184699 | WB,1:1000 |
| **Abcam** | Rabbit anti-Phospho-ERK1/2 | ab201015 | WB,1:1000 |
| **Bioss** | Rabbit anti-ERK5 | bsm-52069R | WB,1:1000 |
| **Bioss**  **ZEN-BIOSCIENCE**  **ZEN-BIOSCIENCE** | Rabbit anti-phospho-ERK5  Anti-Rabbit IgG  Anti-Rabbit IgM | bs-5484R  511203  550110 | WB,1:1000  WB,1:5000  IHC,1:500  WB,1:5000  IHC,1:500 |
